# M28 family peptidase derived from *Peribacillus frigoritolerans* initiates trained immunity to prevent MRSA via the complosome-phosphatidylcholine axis

**DOI:** 10.1080/19490976.2025.2484386

**Published:** 2025-03-30

**Authors:** Cheng-Kai Zhou, Zhen-Zhen Liu, Zi-Ran Peng, Xue-Yue Luo, Xiao-Mei Zhang, Jian-Gang Zhang, Liang Zhang, Wei Chen, Yong-Jun Yang

**Affiliations:** Department of Preventive Veterinary Medicine, College of Veterinary Medicine, Jilin University, Changchun Jilin, P. R China

**Keywords:** MRSA, *Peribacillus frigoritolerans*, M28 family peptidase, trained immunity, complosome, phosphatidylcholine

## Abstract

Methicillin-resistant *Staphylococcus aureus* (MRSA) represents a major global health threat due to its resistance to conventional antibiotics. The commensal microbiota maintains a symbiotic relationship with the host, playing essential roles in metabolism, energy regulation, immune modulation, and pathogen control. Mammals harbor a wide range of commensal bacteria capable of producing unique metabolites with potential therapeutic properties. This study demonstrated that M28 family peptidase (M28), derived from commensal bacteria *Peribacillus frigoritolerans* (*P. f*), provided protective effects against MRSA-induced pneumonia. M28 enhanced the phagocytosis and bactericidal activity of macrophages by inducing trained immunity. RNA sequencing and metabolomic analyses identified the CFB-C3a-C3aR-HIF-1α axis-mediated phosphatidylcholine accumulation as the key mechanism for M28-induced trained immunity. Phosphatidylcholine, like M28, also induced trained immunity. To enhance M28-mediated therapeutic potential, it was encapsulated in liposomes (M28-LNPs), which exhibited superior immune-stimulating properties compared to M28 alone. In vivo experiments revealed that M28-LNPs significantly reduced bacterial loads and lung damage following MRSA infection, which also provided enhanced protection against *Klebsiella pneumoniae* and *Candida albicans*. We first confirmed a link between complement activation and trained immunity, offering valuable insights into the treatment and prevention of complement-related autoimmune diseases.

## GRAPHICAL ABSTRACT

**Figure:**
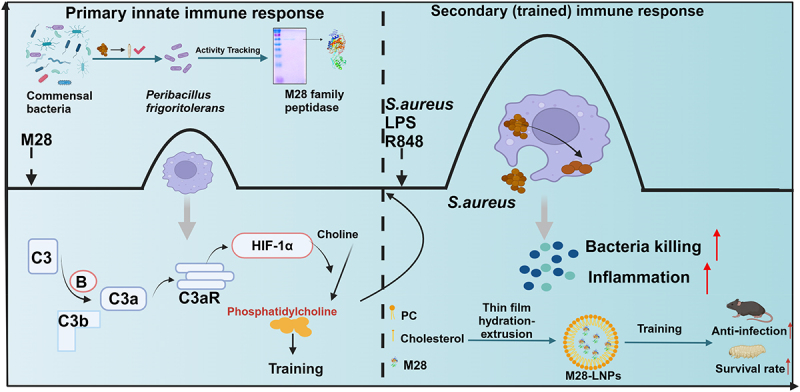

## Introduction

*Staphylococcus aureus* (*S. aureus*) widely infects both humans and animals and is an important pathogen in human and veterinary diseases, seriously affecting human health and the development of the animal industry.^1–3^ It can cause minor skin or soft tissue infections, or even more serious cases such as necrotizing pneumonia, bacteremia, endocarditis, and osteomyelitis. The misuse of broad-spectrum antibiotics has led to the emergence of antibiotic-resistant superbugs, such as methicillin-resistant *S. aureus* (MRSA). Vancomycin is currently the main drug against MRSA, but MRSA strains with reduced susceptibility to vancomycin (hVISA) have been isolated clinically. MRSA has become an increasingly important pathogen in hospitals, communities, and livestock.^1,4^ MRSA is a serious threat to public health and the development of livestock farming, and the World Health Organization (WHO) has prioritized its control. Currently, there is no effective vaccine for *S. aureus*, and with the emergence of drug-resistant strains and the broadening spectrum of resistance, the need for new preventive and therapeutic strategies has become more urgent.

Traditionally, the immune system is classified into innate and adaptive immunity.^5,6^ Adaptive immunity is characterized by the targeted killing process with regulation and memory for host defense against pathogenic infection. It is widely believed that, unlike adaptive immunity, innate immunity lacks immunological memory^6^; however, this view has recently been challenged. Studies have revealed that innate immunity in invertebrate organisms (those without adaptive immune responses) and plants can confer resistance to reinfections.^7^ Moreover, innate immune cells could exhibit memory-like phenotypes and adaptation to homologous or heterologous reinfection when exposed to certain stimuli. This phenomenon is referred to as “trained immunity” or “innate immune memory”.^8,9^ Trained immunity is orchestrated by epigenetic and metabolic reprogramming.^10^ It also promotes hematopoiesis and inhibits tumor growth and metastasis.^11–14^ Research on trained immunity is expected to fundamentally reshape our understanding of immunological memory and host defense, opening new avenues for the prevention and treatment of infectious diseases and other related conditions.

The inducers of trained immunity are mainly classified into two groups, endogenous and exogenous categories. Endogenous inducers mainly include cytokines (IL-1α, IL-1β, and IL-36γ),^15,16^ metabolic products (such as lactate and β-hydroxybutyrate),^17^ oxidized low-density lipoprotein (oxLDL), uric acid crystals, and heat shock proteins.^18–20^ Exogenous inducers are primarily derived from microorganisms and their related components, such as *Limosilactobacillus reuteri*, *Leishmania braziliensis*, β-glucan, BCG, and *Candida albicans*.^21–25^ Exogenous substances exhibit stronger inductive activity compared to endogenous substances. Commensal microbiota are widely colonized on the skin, respiratory tract, gastrointestinal tract, and urogenital tract, with a microbiota structure characterized by diversity and dynamism. Commensal microbiota engage in a mutualistic relationship with the host, which involves host metabolism, energy regulation, immune modulation, and control of pathogenic infections. They are indispensable for maintaining homeostasis and promoting long-term health in the host.^26^ *Peribacillus frigoritolerans* (*P. f*), formerly known as *Brevibacterium frigoritolerans*, is a Gram-positive bacterium. Various strains of *P. f* were previously demonstrated to have numerous beneficial effects, particularly in plants, where they promote growth and protect against pests and diseases.^27–29^ However, the specific activity of *P. f* and its metabolites in animal systems remains largely unexplored.

In this study, we screened *P. f* from a custom-built commensal bacteria library derived from cattle and sheep, which demonstrated protective effects against lethal *S. aureus* infection. Through bioassay-guided isolation, we identified the M28 as the key effector molecule. M28 enhanced resistance against *S. aureus* by inducing trained immunity. Additionally, we found that lipid nanoparticles encapsulating M28 exhibited stronger innate immune-enhancing properties.

## Methods

### Mice

C57BL/6J mice were purchased from Liaoning Changsheng Biotechnology Co., Ltd. The mice were housed in pathogen-free conditions with sterilized food and water and maintained on a 12 hours light/dark cycle. Age- and sex-matched mice (6–8 weeks old) were used for all the experiments.

### Bacteria

*Peribacillus frigoritolerans* (*P. f*， GO-2) was isolated from the gut microbiota of sheep. The other bacteria used for screening are derived from a bacterial library (composed of the gut microbiota from cattle and sheep). Methicillin-resistant *Staphylococcus aureus* (MRSA) (USA300, TCH1516), *Klebsiella pneumoniae* (ATCC 13883), *Candida albicans* (BNCC337321), and *Escherichia coli* (DH5α), and *Escherichia coli* (BL21) were preserved in our laboratory.

### Bacterial screening

The protective effect of the bacteria was evaluated by statistically analyzing the survival rate of the *Galleria mellonella* larvae over 48 hours following *S. aureus* infection. The specific approaches are as follows: The *Galleria mellonella* larvae, approximately 2–2.5 cm in length and 200–250 mg in weight, were purchased from Tianjin Huiyode Biotechnology Co., Ltd. (Tianjin, China). In order to screen for target bacteria, several bacteria (sourced from our laboratory’s self-established bacterial libraries) were cultured to the logarithmic phase, harvested by centrifugation, washed twice with PBS, and then resuspended in PBS. The larvae were initially exposed to candidate bacteria (5 × 10^4^ CFU, 10 μL) to the last left pro-leg using a LSP01-2A laboratory microinjection pump. Larvae were incubated at 37°C for 3 days and then injected with a lethal dose of *S. aureus* (5 × 10^5^ CFU). The number of dead larvae was counted every 6 hours over a 48-hour period, and the survival curves were analyzed using the log-rank (Mantel-Cox) test.

### Pneumonia model

Mice were administered with PBS, live-*P. f*, or heat-killed *P. f* (HK-*P. f*) via oral gavage (200 μL, 1 × 10^8^ CFU) once daily for 3 consecutive days. After a 7-day interval, the mice were intranasally infected with *S. aureus* (USA300, 1 × 10^8^ CFU, 20 μL). Lung tissue, bronchoalveolar lavage fluid (BALF), and blood were collected 24 hours post-infection for analysis. Bacterial loads were determined in lung tissue, BALF and blood. The lung tissues were fixed to embed for pathological analysis. In the *P. f* culture cell-free supernatant (CFS) treatment experiments, *P. f* was incubated in TSB medium (HaiBo, QingDao, HB4114) for 12 hours at 37°C in shake flasks. The bacterial solution was centrifuged at 12,000 × *g* for 5 min, and the supernatants were collected. Mice were then gavaged with 100 μL of the CFS, with TSB medium serving as the control. In the M28 treatment experiment, mice received an intraperitoneal injection of M28 (4 mg/kg, 100 μL), with PBS as the control. For the liposome experiment, mice were injected via the tail vein with PBS (100 μL), M28 (4 mg/kg, 100 μL) or Lipo (100 μL), M28-Lipo (4 mg/kg, 100 μL).^30^

### Lung histopathology score

Each lung tissue section was examined under an optical microscope at a magnification of × 200, and the score for each lung was determined by assessing five observation fields. The evaluation included alveolar congestion, hemorrhage, and infiltration or aggregation of inflammatory cells in the alveolar spaces or vascular walls, as well as alveolar wall thickness/formation of hyaline membranes. Each factor was scored on a 5-point scale: 0 = minimal damage, 1 = mild damage, 2 = moderate damage, 3 = severe damage, and 4 = maximal damage. Therefore, the minimum possible score was 0, and the maximum possible score was 16.^31,32^

### Survival experiment

*Galleria mellonella* larvae were used to assess survival following bacterial infection. Bacteria (5 × 10^4^ CFU, 10 μL), CFS (10 μL), M28 (2 mg/mL, 10 μL), and Lipo (10 μL) and M28-Lipo (2 mg/mL, 10 μL) were injected into the legs of the larvae. 3 days later, *S. aureus* (5 × 10^5^ CFU, 10 μL), *Klebsiella pneumoniae* (5 × 10^5^ CFU, 10 μL), and *Candida albicans* (5 × 10^5^ CFU, 10 μL) were injected to evaluate the larvae’s survival rate over a 48-hour period.

## Trained immunity experiment

### In vitro

Peritoneal macrophages (PMs) were isolated from 6-week-old C57BL/6J mice and seeded into 96-well plates (2 × 10^5^ cells/well), 24-well plates (5 × 10^5^ cells/well), or 6-well plates (3 × 10^6^ cells/well). The cells were first stimulated with M28 (5 μg/mL) for 24 hours, with heat-killed *Candida albicans* (HK. *ca*) serving as a positive control. After 24 hours, the cells were washed twice with warm PBS and kept in RPMI 1640 medium (Gibco) supplemented with 10% FBS (Gibco) for 5 days. On day 6, the cells were re-stimulated with PBS, LPS (100 ng/mL), R848 (100 ng/mL), or *S. aureus* (USA300, MOI = 5). For the inhibitor experiments, cells were pre-treated with Iptacopan (MCE, HY-127105), SB290157 (MCE, HY-101502A), or Ascorbate (MACKLIN, A800295, 10 μM), 2-DG (MCE, HY-13966, 5 mM) for 24 hours, followed by the stimulation with M28 (5 μg/mL).^33^

### In vivo

Age- and sex-matched C57BL/6J mice (6 weeks old) were administered with PBS, Live-*P. f*, HK-*P. f* or *E. coil* DH5α and *E. coil* BL21 via oral gavage (200 μL, 1 × 10^8^ CFU) once daily for 3 consecutive days. After a 7-day interval, PMs or alveolar macrophages (AMs) were isolated and seeded into 96-well cell culture plates (2 × 10^5^ cells/well). The cells were then re-stimulated with PBS, LPS (100 ng/mL), or R848 (100 ng/mL). After 24 hours, the supernatants were collected for NO or TNF-α measurement.

C57BL/6J mice were injected via the tail vein with 100 μL of PBS, 4 mg/kg of M28, 100 μL of Lipo, or 4 mg/kg of M28-Lipo. 7 days later, PMs were isolated and seeded into 96-well cell culture plates (2 × 10^5^ cells/well). The cells were then re-stimulated with PBS, LPS (100 ng/mL), or R848 (100 ng/mL). After 24 hours, the supernatants were collected for NO measurement.

## Production and purification of M28 family peptidase

*P. f* was inoculated in TSB medium and grown for 12 hours at 37°C. The cultures were then transferred at 1% (v/v) into 1 L of TSB medium and incubated for an additional 12 hours. The bacterial solution was centrifuged at 12,000 × *g* for 5 min, and the supernatants were collected and filter-sterilized through a 0.22 μm filter. Saturated ammonium sulfate (SAS) solution was applied to precipitate proteins. After overnight stirring at 4°C, the solution was centrifuged at 12,000 × *g* for 10 min. The resulting precipitate was redissolved in sterile distilled water, and the SAS was removed by dialysis using 1 kDa dialysis bags, yielding the crude extract. The crude extract was further purified by anion exchange chromatography, resulting in six fractions (Fr1–Fr6). The active fractions were then loaded onto a Sephadex G-50 gel filtration column (Solarbio, China) pre-equilibrated with sterile distilled water. The column was eluted at a flow rate of 1 mL/min, collecting four fractions (Fr4–1–Fr4–4). Subsequently, the active samples were further fractionated using a HPLC system (PuriMaster-2000, Kezhe, Shanghai) with a Durashell C18 column, yielding six fractions (Fr4-2-1–Fr4-2-6). Among these fractions, Fr4-2-4 was identified as the active component (Figure S4C).

## SDS-PAGE and MALDI-TOF/TOF analysis of M28 family peptidase

The final active fractions were separated using 12% SDS-PAGE at 80 V for 20 min and then at 120 V for 1 hours. Then, the gel was stained using the Coomassie brilliant blue staining method to determine the molecular mass. The protein bands were excised and sent to Sangon Biotech (Shanghai, China) for protein sequencing. The protein sequences were then analyzed using MALDI-TOF/TOF mass spectrometry (Shimadzu, Japan).

## RNA isolation and real-time PCR measurement

Total RNA was isolated from cultured cells using RNAiso Plus (Takara, Japan) and cDNA was synthesized through reverse transcription using a Reverse Transcription Kit (Yamei, China). Quantitative RT-PCR (Applied Biosystems, USA) was then performed with SYBR Green (Roche, Switzerland) and primers obtained from primer bank and Sangon Biotech (primer sequences are detailed in Table S1). β-actin was used as an internal reference. Relative gene expression was calculated using the 2^−ΔΔCT^ method.

## ELISA

Supernatants from PMs cultures were collected for ELISA. The experiment was conducted according to the manufacturer’s protocol for the kits. Mouse TNF-α ELISA kits were purchased from R&D Systems (DY410).

## Nitric oxide (NO) production

NO production in cell culture supernatants was measured by the Griess method and according to the indications provided with the NO assay kit (Beyotime, China, S0021S).

## Western blotting

PMs were lysed with RIPA buffer containing protease and phosphatase inhibitors. The protein samples were denatured at 100°C for 5 min and loaded onto 12% SDS-PAGE gels. Following electrophoresis, the proteins were transferred to a polyvinylidene difluoride (PVDF) membrane. The PVDF membranes were blocked at room temperature for 2 hours with 5% nonfat dry milk in TBST. After blocking, the membranes were incubated overnight at 4°C with primary antibodies (p-mTOR, Proteintech #67778–1, 1:2000; p-Akt, Proteintech #66444–1, 1:2000; HIF-1α, Wanleibio #01607, 1:2000; β-actin, CST #4967, 1:10000). The membranes were subsequently washed four times with TBST. Subsequently, the membranes were incubated with secondary antibodies (HRP-conjugated Goat Anti-Rabbit IgG (H+L), Proteintech #SA00001–2, 1:5000) diluted in TBST at room temperature for 2 hours. Finally, the membranes were treated with enhanced chemiluminescence (ECL) reagent (Thermo, USA), and the bands were visualized using the ChemiDoc MP Imaging System (Tanon, China).

## Phagocytosis and intracellular killing bacteria assay

M28-trained or untrained PMs were infected with *S. aureus* (USA300, MOI = 5) for 30 min in RPMI 1640 at 37°C. After infection, the cells were washed twice with RPMI 1640 to remove non-phagocytosed bacteria. Medium supplemented with 100 μg/mL gentamicin, 200 U/mL penicillin, and 200 μg/mL streptomycin was added to each well, and the cells were incubated at 37°C. After an additional 30 min, PMs were washed and lysed with 0.1% Triton X-100 to release intracellular *S. aureus*. The lysates were then plated on TSB agar and incubated at 37°C overnight. Colonies were counted for statistical analysis. Killing efficiency was calculated as ([CFU/mL at 3 or 6 hours])/(CFU/mL at 1 hour).^23,34^

## RNA sequencing (RNA-Seq)

PMs were treated with PBS or M28 (5 μg/mL) for 24 hours. After treatment, the cells were washed with PBS and allowed to rest for 3 days. Total RNA was then extracted from the cultured cells using RNAiso Plus. The library was evaluated for quantification using Qubit and real-time PCR, as well as for size distribution analysis on a bioanalyzer. Clustering of the index-coded samples was performed using the TruSeq PE Cluster Kit v3-cBot-HS on Illumina. After cluster generation, library preparations were sequenced on an Illumina Novaseq platform, generating 150 bp paired-end reads. For differential gene expression analysis, the read counts for each sequenced library were normalized using a scaling factor via the edgeR package. Differential expression analysis was performed for both samples using the edgeR (3.22.5) R package. P-values were adjusted using q-values, with thresholds set at q < 0.005 and |log2(FoldChange)| > 1 for significant differential expression.

## Non-targeted metabolomics analysis

PMs were treated with PBS or M28 (5 μg/mL) for 24 hours. Following treatment, the cells were washed with PBS and allowed to rest for 5 days. Cells were trypsinized, and live cells were counted. The samples were placed in the EP tubes and resuspended with prechilled 80% methanol by well vortex. Then, the samples were melted on ice and whirled for 30 s. After the sonification for 6 min, they were centrifuged at 3500 × *g*, 4°C for 1 min. The supernatant was freeze-dried and dissolved with 10% methanol. Finally, the samples were injected into the UHPLC-MS/MS system (ThermoFisher, Germany) for analysis. Samples were injected onto a Hypersil Gold column (100 × 2.1 mm, 1.9 μm) using a 12-min linear gradient at a flow rate of 0.2 mL/min. The eluents for the positive and negative polarity modes were eluent A (0.1% FA in water) and eluent B (methanol). The solvent gradient was set as follows: 2% B, 1.5 min; 2–85% B, 3 min; 85–100% B, 10 min; 100–2% B, 10.1 min; 2% B, 12 min. Q ExactiveTM HF mass spectrometer was operated in positive/negative polarity mode with a spray voltage of 3.5 kV, a capillary temperature of 320°C, a sheath gas flow rate of 35 psi, an aux gas flow rate of 10 L/min, an S-lens RF level of 60, an Aux gas heater temperature of 350°C. The raw data files generated by UHPLC-MS/MS were processed using the Compound Discoverer 3.3 (CD3.3, ThermoFisher) to perform peak alignment, peak picking, and quantitation for each metabolite. These metabolites were annotated using the KEGG database, HMDB database, and LIPIDMaps database. The metabolites with VIP > 1 and P-value < 0.05 and fold change ≥ 2 or FC ≤ 0.5 were considered to be differential metabolites.

## Phosphatidylcholine assay

PMs were pre-treated with iptacopan (10 μM) or SB290157 (10 μM) for 24 hours, followed by the stimulation with M28 (5 μg/mL). After 24 hours, the cells were washed twice with warm PBS and kept in RPMI 1640 medium (Gibco) supplemented with 10% FBS (Gibco) for 5 days. Cells were collected for subsequent assays. Phosphatidylcholine concentration was determined using a phosphatidylcholine assay kit (Abcam, UK, ab83377). The experiment was conducted according to the manufacturer’s protocol.

## Preparation and characterization of the liposome

Liposomes were prepared using the thin-film hydration and extrusion method. Phosphatidylcholine (MACKLIN, L812366, 10 μM) and cholesterol (MACKLIN, C804517, 5 μM) were dissolved in 2 mL of chloroform, which was then transferred to a 20 mL vial. Chloroform was evaporated under vacuum using a rotary evaporator to form a thin lipid film. Subsequently, M28 (5 mg/mL, 1 mL) was added and thoroughly mixed by rotation for 1 hour to facilitate the elution of the lipid film, resulting in the formation of liposomes. The liposomes were then extruded 21 times through polycarbonate membranes with pore sizes of 400 nm, 200 nm, and 50 nm in sequence. The resulting mixtures were transferred to a 100 kDa MWCO Vivaspin tube and centrifuged at 3,500 × *g* at 4°C until approximately 1 mL of volume remained. Next, PBS was added to the Vivaspin tube, and centrifugation was carried out at 3,500 × *g* for 10 min. This step was repeated twice to remove any unbound M28. Finally, the liposomes were filtered through a 0.22 μm filter to obtain a clarified solution.

## Transmission electron microscopy (TEM)

Drops of the liposome suspension were placed onto a carbon-coated copper grid and allowed to dry. The samples were then stained with a 2% phosphotungstic acid solution for 5 min at room temperature and subsequently examined under TEM (Tokyo, Japan).

## Dynamic light scattering (DLS)

The hydrodynamic diameter and zeta potential of the as-prepared liposome were measured using performing DLS characterizations (Malvern, UK).

## Encapsulation efficiency and loading capacity

M28-Lipo was added to 400 μL of methanol, vortexed for 10 min, sonicated for 20 min, and then centrifuged at 13,500 × *g* for 15 min. The concentration of M28 in the supernatant was determined spectrophotometrically (OD_280 nm_), allowing for the calculation of the drug concentration in the liposomes. The drug loading and encapsulation efficiency were then calculated as follows:

Encapsulation eficiency (%) = (the loaded drug mass)/(the total drug mass) × 100%. Loading capacity (%) = (the loaded drug mass)/(the loaded drug mass + the total mass of liposome) × 100%.^35^

## In vitro release

The M28-Lipo was placed in a dialysis bag (100 kDa) and immersed in PBS containing 10% FBS at 37°C. Agitation was maintained for 24 hours. At predetermined time points (0, 1, 2, 4, 6, 8, 12, and 24 hour), samples were collected from the dialyzate, and the concentration of M28 was determined using the previously described method.

## Statistical analysis

Statistical analyses were performed using GraphPad Prism software version 8.0 (GraphPad Inc., La Jolla, CA). Data are shown as mean values ± SEM. Data were analyzed using a T-test for comparisons between two groups or one-way analysis of variance (ANOVA), and survival assays were analyzed using the log-rank (Mantel–Cox) tests, as indicated in the figure legends. P-values of less than 0.05 were considered statistically significant, with the following significance levels: *p < 0.05; **p < 0.01; ***p < 0.001; ****p < 0.0001; ns, not significant.

## Results

### *Pre-exposure of mice and macrophages to live* P. f *enhances bacterial killing and increases pro-inflammatory responses*

**Figure 1.**
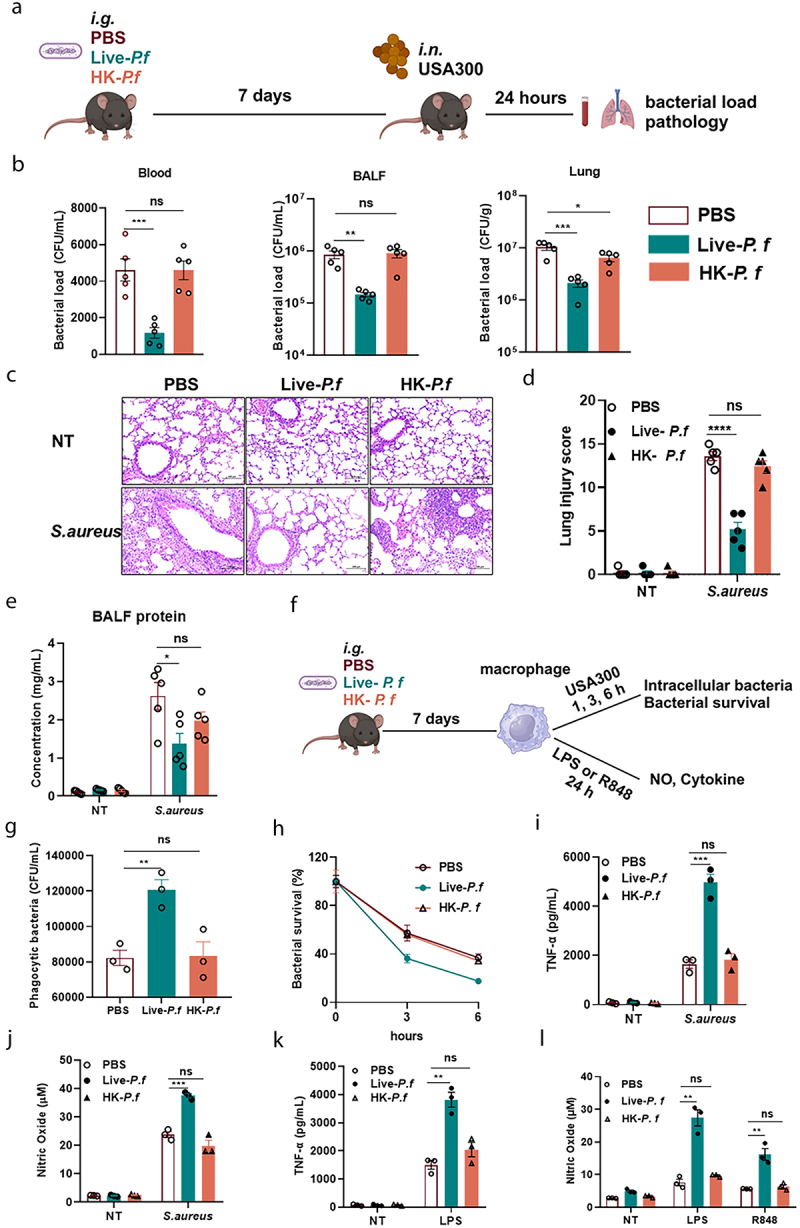
Live *P. f* protects against *S. aureus* pneumonia.

We screened custom-built commensal bacterial libraries and we locked into a strain of *P. f* (Figure S1A-E and Figure S2A-C). We found that *Galleria mellonlla* larva pre-exposed to live-*P. f* showed a higher survival rate than that of the PBS-pretreated and HK-*P. f* pretreated groups (Figure S2D). Subsequently, we treated mice with PBS, Live- *P. f*, and HK- *P. f*，7 days later， the mice were intranasally infected with *S. aureus* (Figure 1(a)). After 24 hours, bacterial loads in blood, lung tissues, and BALF were significantly lower in the live-*P. f* group compared to the PBS group, whereas no significant change was observed in the HK-*P. f* group (Figure 1(b)). We also evaluated the histopathologic damage in the lungs following *S. aureus* infection. The lung tissues showed obvious damage with hemorrhage, edema, and inflammatory cell infiltration in the PBS group, the live-*P. f* treated group significantly attenuated these symptoms, whereas HK-*P. f* failed to relieve these pathologic changes (Figure 1(c,d)). The protein leakage levels of the lungs showed similar trends (Figure 1(e)). These findings suggested that live *P. f*, but not dead *P. f*, promoted bacterial clearance and attenuated organ damage after *S. aureus* infection.

(a) Schematic of the experimental model in mice. Mice were gavaged with PBS, live-*P. f*, or HK- *P. f* (1 × 10^8^CFU, 200 µL) administered once daily for three consecutive days. 7 days later, the mice were intranasally challenged with *S. aureus* (1 × 10^8^ CFU, 20 µL). 24 hours post-infection, blood, bronchoalveolar lavage fluid (BALF), and lung tissue were collected for analysis (PBS group *n* = 5; live-*P. f* group *n* = 5; HK-*P. f* group *n* = 5). (b) Bacterial load in blood, BALF, and lung tissue (*n* = 5 for each group). (c) Representative H&E staining images of lung tissue from mice pretreated with PBS, Live-*P. f*, or HK-*P. f* (scale bar: 100 μm, magnification: ×200). (d) Lung pathology scores (*n* = 5 for each group). (e) Exudative protein concentration in BALF (*n* = 5 for each group). (f) Mice were gavaged with PBS, Live-*P. f*, or HK- *P. f* (1 × 10^8^CFU, 200 µL) administered once daily for three consecutive days. 7 days later, macrophages were isolated from the peritoneal cavity and infected with *S. aureus* at a multiplicity of infection (MOI = 5), Phagocytosis (g) and intracellular survival (h) of *S. aureus* by macrophages were assessed at 1 hour or 3 and 6 hours post reinfection (*n* = 3), supernatants were collected to measure TNF-α (i) and NO (j) levels. Alternatively, macrophages were stimulated with LPS (100 ng/mL) and R848 (100 ng/mL) for 24 hours. Supernatants were collected to measure TNF-α (k) and NO (l) levels. Data were presented as means ± SEM. One-Way and Two-way ANOVA with Tukey’s multiple comparisons tests, and unpaired Student’s t tests were performed. *ns*, not significant, *P < 0.05, **P < 0.01, ***P < 0.001.

To further investigate whether the protective effect of immune cells was enhanced by live- *P. f* pretreatment, we performed further analysis. We treated mice with PBS, live- *P. f*, and HK- *P. f*, after 7 days, isolated peritoneal macrophages were re-infected with *S. aureus* (Figure 1(f)). Our results showed that the live-*P. f* pretreatment enhanced the phagocytosis and killing capacity of macrophages challenged with *S. aureus* (Figure 1(g,h)). It was also found that the levels of nitric oxide (NO) and TNF-α released from macrophages were significantly enhanced after treatment with *S. aureus* and LPS (a ligand for TLR4, is a classical and potent agonist of innate immune responses), R848 (a ligand for TLR7/8, can be used to mimic single-stranded RNA viruses) in the live-*P. f* group (Figure 1(i-l)). AMs play a critical role in defending against pulmonary infections. We treated mice with PBS, *P. f*, and low virulence *Escherichia coli* (*E. coli* DH5α and BL21) which was used as a control. After 7 days, AMs were isolated and re-infected with *S. aureus* (Figure S3A). The results showed that the phagocytic and bactericidal capabilities against *S. aureus* were significantly enhanced in the *P. f* group compared to the PBS and *E. coli* control groups (Figure S3B, C). Taken together, these findings clearly showed that pretreatment with live-*P. f* enhanced the host immune response and effectively protected against *S. aureus* infection.

### *M28 family peptidase is the main functional molecule produced by* P. f

**Figure 2.**
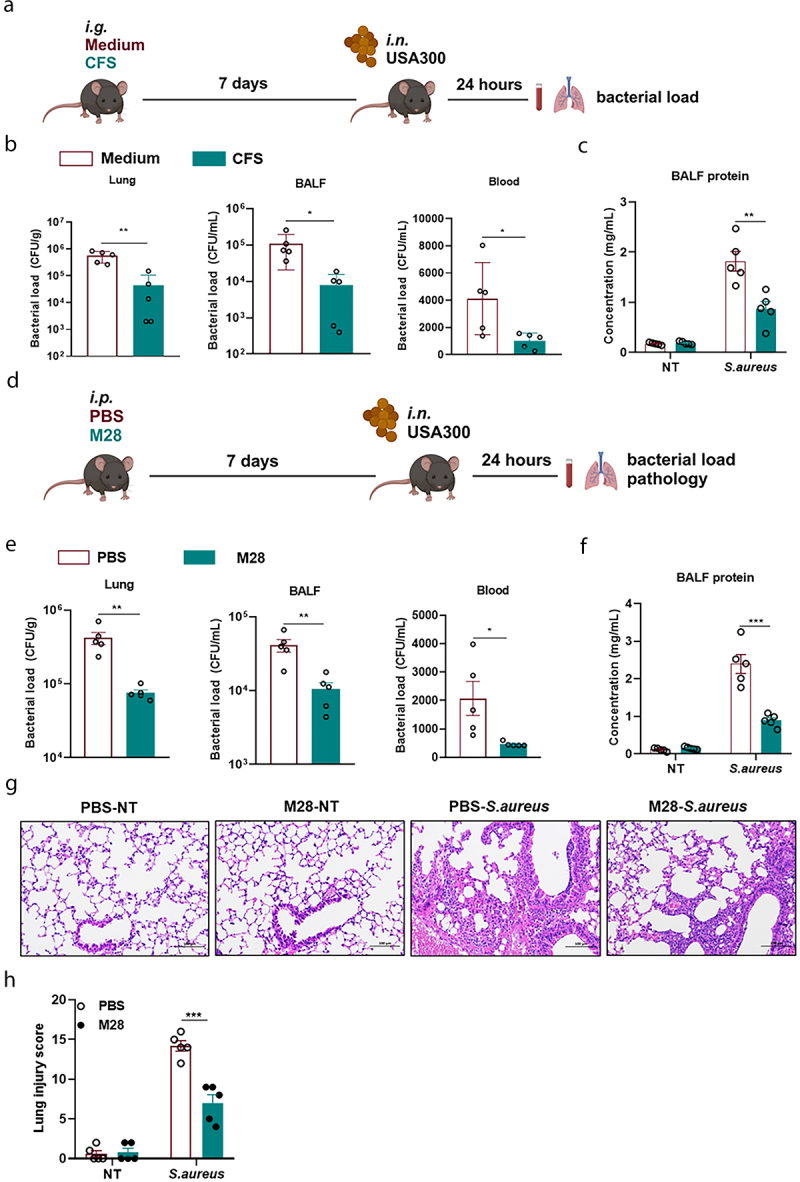
M28 family peptidase is the main functional molecule produced by *P. f.*

Live *P. f* pretreatment was efficient for host defense against *S. aureus* infection, whereas dead *P. f* was not, indicating that a substance derived from living bacteria played a key role in the bacteria-dependent protective effect. Consequently, we investigated whether cell-free supernatant (CFS) from live-*P. f* could enhance the organism’s resistance to *S. aureus* infection (Figure 2(a)). As expected, mice pretreated with *P. f* CFS exhibited a significant reduction in bacterial load in lung tissues, BALF, and blood compared to the control group, as well as decreased protein leakage levels in lung tissues (Figure 2(b, c)). Similarly, in the *Galleria mellonella* model, the CFS group also showed increased survival rates compared to the control group after *S. aureus* infection (Figure S2E and Figure S4A). These findings suggested that live *P. f* could produce a functional substance that conferred protection against *S. aureus* infection.

(a) *P. f* was incubated in TSB medium for 12 hours at 37°C in shake flasks. Subsequently, mice were then gavaged with 100 μL of the cell-free supernatant (CFS), while TSB medium (100 μL) served as the control. 7 days later, the mice were intranasally challenged with *S. aureus* (1 × 10^8^ CFU, 20 μL). 24 hours post-infection, blood, BALF, and lung tissue were collected for analysis (*n* = 5 for each group). (b) Bacterial load in lung tissue, BALF, and blood (*n* = 5 for each group). (c) Exudative protein concentration in BALF (*n* = 5 for each group). (d) Mice were injected intraperitoneally with PBS or M28 (4 mg/kg, 100 μL), and 7 days later, they were infected intranasally with *S. aureus* (1 × 10^8^ CFU, 20 μL). 24 hours post-infection, blood, BALF, and lung tissue were collected for analysis (*n* = 5 for each group). (e) Bacterial load in lung tissue, BALF, and blood (*n* = 5 for each group). (f) Exudative protein concentration in BALF (*n* = 5 for each group). (g) Representative H&E staining images of lung tissues from PBS and M28-pretreated mice (scale bar: 100 μm, magnification: ×200). (h) Lung pathology scores (*n* = 5 for each group). Data were presented as means ± SEM. One-way and two-way ANOVA with Tukey’s multiple comparison tests and unpaired Student’s t tests were performed. *P < 0.05, **P < 0.01, ***P < 0.001.

To further investigate the nature of the protective substances secreted by live-*P. f*, we isolated and purified the substances using activity-based tracking. A detailed process was illustrated by a flowchart (Figure S4C). Exposure of the CFS to proteinase K and trypsin resulted in a complete loss of activity, suggesting that the active substance was of a proteinaceous nature (Figure S4B). Initial fractionation of the bacterial CFS was achieved through ammonium sulfate precipitation, followed by anion exchange chromatography, dextran gel G50 filtration, and preparative HPLC for further purification. The purity of the final product, Fr4-2-4, was assessed by analytical HPLC, and SDS-PAGE analysis revealed a molecular weight of approximately 55 kDa. MALDI-TOF/TOF mass spectrometry identified the protein as a member of the M28 family peptidase (GenBank: WP_239860088.1) (Figure S4D-L).

Further, we pretreated mice with PBS or M28, followed by a nasal infection with *S. aureus* after 7 days (Figure 2(d)). The results showed that the bacterial loads in lung tissues, BALF, and blood were significantly lower in the M28 group (Figure 2(e)). Meanwhile, lung injury was significantly attenuated in the M28-treated mice. Compared to PBS, M28 treatment reduced pulmonary hemorrhage, protein leakage, and inflammatory cell infiltration, among others (Figure 2(f-h)). Moreover, the survival rates were also significantly improved (p ＜ 0.01) in the M28-pretreatment group after infection with *S. aureus* (Figure S5A). The results indicated that M28 enhanced the innate immune response of *Galleria mellonella* larvae, leading to a higher survival rate upon infection with *S. aureus*. Collectively, these data indicated that M28 family peptidase was the major effector of *P. f* in host defense against *S. aureus* infection.

### M28 imprints innate memory-like responses in macrophages

**Figure 3.**
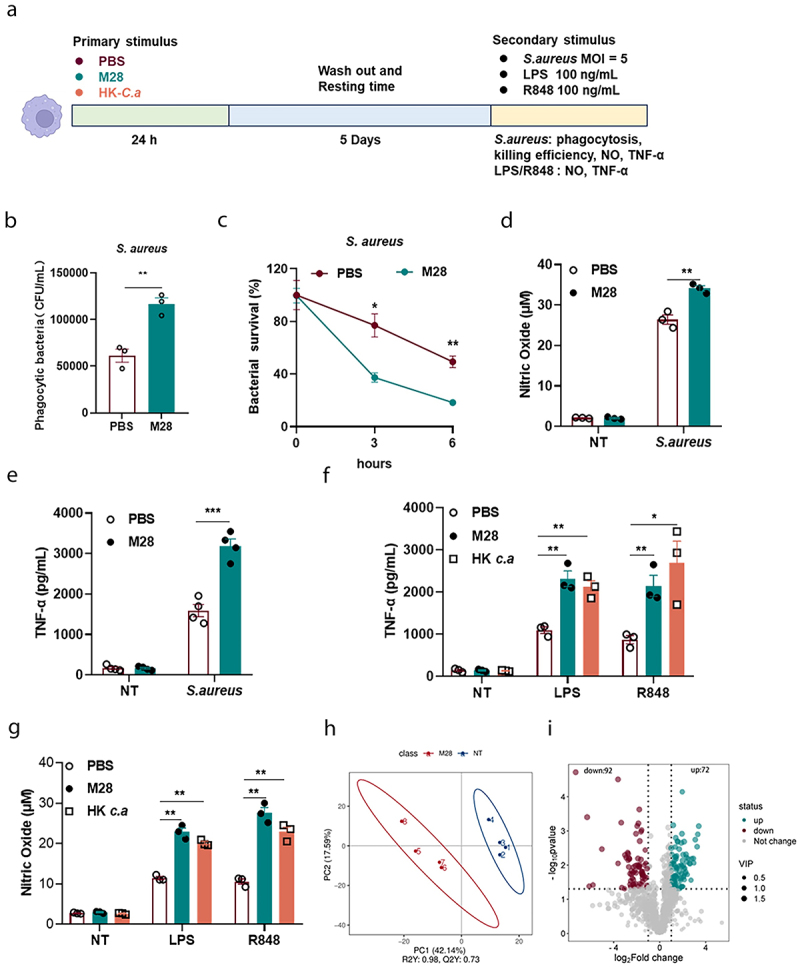
M28 protects against *S. aureus* infection by inducing trained immunity.

Innate immune memory or trained immunity refers to the phenomenon in which innate immune cells undergo epigenetic and metabolic reprogramming following stimulation, resulting in a stronger nonspecific immune response upon re-exposure to homogeneous or heterogeneous stimulus.^10,33^ To determine whether M28 confers its protective effects by inducing trained immunity, we used a trained immunity model in macrophages (Figure 3(a)). Heat-killed *Candida albicans* (HK *C. a*) and its main active component β-glucan were well-described stimuli to induce trained immunity and thus were used as positive controls.^36,37^ PAMP molecules (such as LPS and R848) are generally shown as the classical secondary stimulations used to induce innate immune responses.^17,36,38^ Our findings revealed that M28-trained macrophages exhibited increased bacterial phagocytosis and intracellular bacterial killing capacity, as well as enhanced release of the inflammatory mediator NO and TNF-α (Figure 3(b-e)). Consistent with the above results, the same tendency was also observed upon stimulation with the LPS and R848, and the extent of released inflammatory mediators in the M28-trained group was comparable to induction observed by positive control (HK *C. a*). nearly to the extent observed in the HK *C. a* training group (Figure 3(f,g)). These results indicated that M28-trained macrophages exhibited a more robust immune response upon secondary stimulation. As expected, it also enhanced survival rates of *Galleria mellonella* after infection with *Klebsiella pneumoniae* and *Candida albicans* (Figure S5B, C). Additionally, we assessed metabolic changes and discovered that M28-trained macrophages had distinct metabolite profiles compared to PBS-trained macrophages, with a total of 164 differential metabolites (72 up-regulated and 92 down-regulated) (Figure 3(h,i)). In summary, these findings provided substantial evidence that M28 induced trained immunity accompanied by metabolic changes and enhanced immune function.

(a) Schematic diagram of in vitro cellular experiments for the trained immunity models. Macrophages were pretreated with PBS or M28 (5 µg/mL) for 24 hours. HK. *C a* was used as a positive control. After 24 hours, the cells were washed twice with PBS to remove M28 and continued incubation was performed. After 5 days, the macrophages were infected with *S. aureus* (MOI = 5). Phagocytosis of *S. aureus* (b) and intracellular viability (c) was assessed at 1 and 3 and 6 hours post-reinfection (*n* = 3). Supernatants were collected to measure NO (d) and TNF-α (e) levels (*n* = 3 or 4). Alternatively, macrophages were stimulated with LPS (100 ng/mL) and R848 (100 ng/mL) for 24 hours. Supernatants were collected to measure TNF-α (f) and NO (g) levels (*n* = 3). Schematic diagram of in vitro cellular experiments for the trained immunity models. Macrophages were pretreated with PBS or M28 (5 µg/mL) for 24 hours. After 24 hours, the cells were washed twice with PBS to remove M28 and continued incubation was performed. After 5 days, macrophages were lysed for non-target metabolomic assays. (h) Scatter plots of PLS-DA of non-targeted metabolomics analysis from PBS and M28 (*n* = 4). (i) Volcano plot of non-targeted metabolomics analysis (M28 group vs PBS group). Data were presented as means ± SEM. One-way ANOVA and two-way ANOVA with Tukey’s multiple comparison tests, and unpaired Student’s t tests were performed. *P < 0.05, **P < 0.01, ***P < 0.001.

### HIF-1α, but not the process of glycolysis, is essential for M28-induced trained immunity

**Figure 4.**
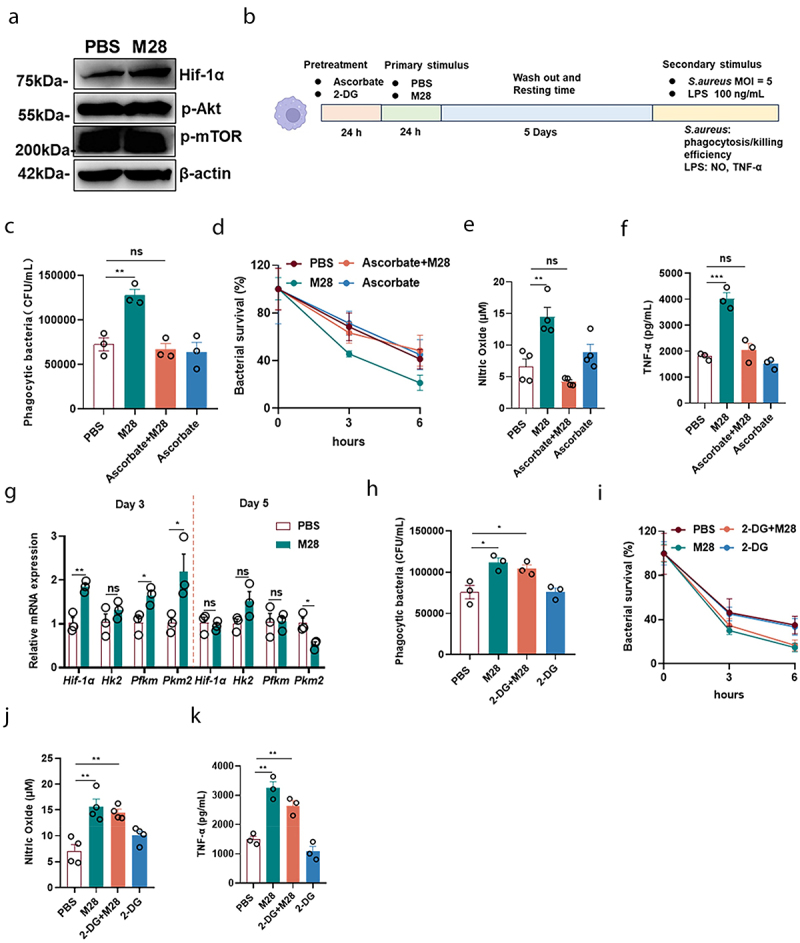
The activation of HIF-1α signaling by M28 triggers trained immunity.

AKT-mTOR-HIF-1α-dependent metabolic reprogramming from oxidative phosphorylation to aerobic glycolysis is a well-established mechanism for β-glucan-induced trained immunity.^33^ To further explore the mechanism of M28-induced trained immunity, we first examined the activation of the AKT-mTOR-HIF-1α signaling pathway in M28-trained macrophages. In our study, we were surprised to find that the levels of p-AKT and p-mTOR were not significantly changed after M28 training. Unexpectedly, HIF-1α protein levels were significantly higher in M28-trained macrophages compared to the control group (Figure 4(a)). Furthermore, pharmacological inhibition of HIF-1α attenuated M28-induced trained immunity, as evidenced by the diminished phagocytic and bactericidal activity against *S. aureus*, along with the reduced release of NO and TNF-α (Figure 4b-f). HIF-1α, a key metabolic regulator, promotes glycolysis and upregulates the expression of glucose transporters.^39^ Subsequently, we performed qRT-PCR to assess the expression of key glycolytic enzymes (*Hk2, Pfkm, and Pkm2*) on days 3 and 5 post-treatment. Although the mRNA levels of these enzymes were increased on day 3 but showed no significant changes by day 5 (Figure 4(g)). Interestingly, inhibition of glycolysis with 2-DG did not influence M28-induced trained immunity (Figure 4(h-k)). These results suggested that while HIF-1α played the key role in M28-triggered trained immunity, whereas the glycolysis process might not be practically important.

(a) After stimulation with M28 (5 µg/mL) for 24 hours, M28 was removed, and cell lysates were collected 3 days later. Western blot analysis was performed to assess the levels of *p*- AKT, *p*- mTOR, and HIF-1α. (b) For the inhibitor assay, cells were treated with ascorbate (HIF-1α inhibitor, 10 µM, pretreated for 24 hours) or 2-DG (glycolysis inhibitor, 5 mM, pretreated for 24 hours), followed by treatment with M28 (5 µg/mL) for 24 hours. After this period, the cells were washed twice with PBS to remove M28, and continued incubation was performed. After 5 days, the macrophages were infected with *S. aureus* (MOI = 5). Phagocytosis of *S. aureus* (c, h) and intracellular killing (d, i) was assessed at 1 and 3 and 6 hours post-reinfection (n = 3). Alternatively, macrophages were stimulated with LPS (100 ng/mL) for 24 hours. Supernatants were collected to measure the levels of NO (e, j) and TNF-α (f, k) (n = 3 or 4). (g) The mRNA levels of key enzymes of glycolysis at 3 and 5 days (n = 3). Data were presented as means ± SEM. One-way ANOVA and two-way ANOVA with Tukey’s multiple comparison tests, and unpaired Student’s t tests were performed. *ns*, not significant, *P < 0.05, **P < 0.01, ***P < 0.001.

### M28 induces trained immunity via the CFB-C3a-C3aR-HIF-1α signaling pathway

**Figure 5.**
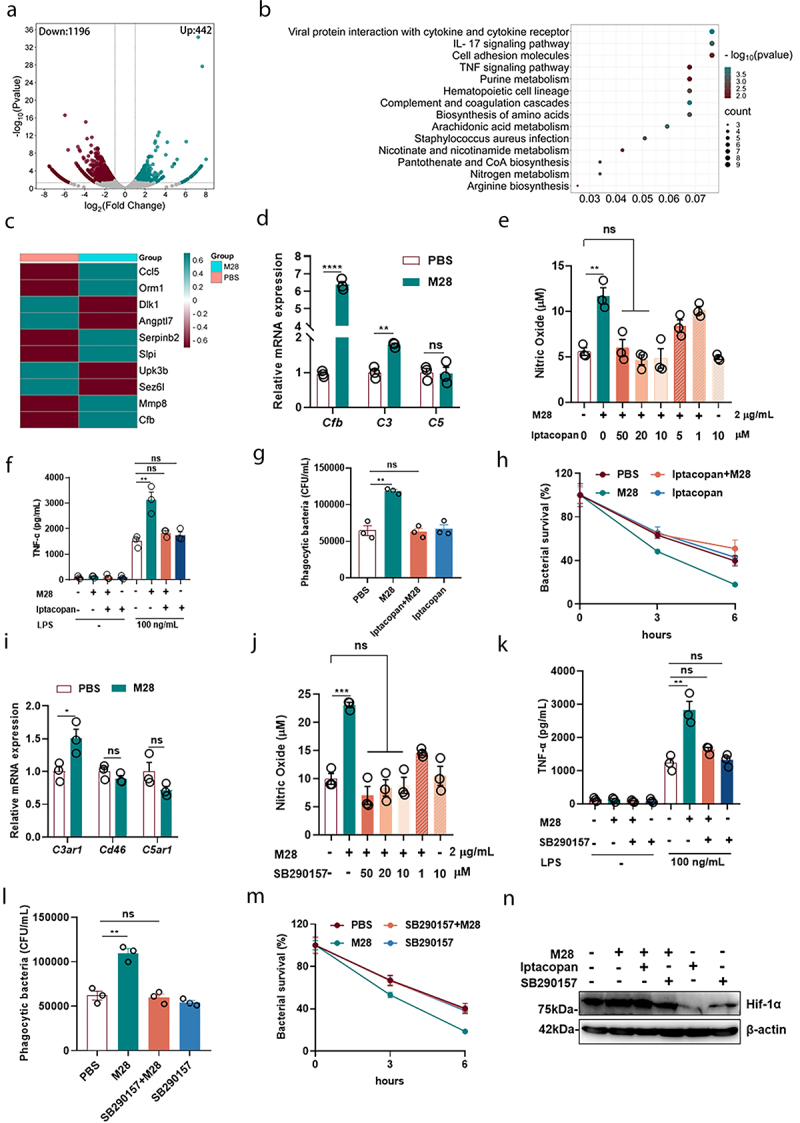
M28 induces trained immunity via the CFB-C3a-C3aR-HIF-1α signaling pathway.

To further investigate the mechanistic basis of M28-induced trained immunity, we conducted RNA-seq on macrophages from both M28-trained and PBS-trained groups. RNA-seq analysis revealed a significant number of differentially expressed genes (DEGs) in M28-trained macrophages compared to PBS controls, identifying a total of 1,638 DEGs, including 442 upregulated and 1,196 downregulated genes (Figure 5(a)). KEGG pathway analysis highlighted several pathways associated with innate immune responses, including cytokine secretion, complement and coagulation cascades, IL-17 signaling, TNF signaling, *Staphylococcus aureus* infection, and metabolic pathways (Figure 5(b)). In conjunction with our metabolic analysis of M28-trained macrophages, a large number of differential metabolites were revealed (Figure 3(h, i)). In the RNA-seq data, we observed significant activation of the complement and coagulation cascades (*p* value = 0.000087). Studies have demonstrated that the complement system plays an important role in cellular metabolic activities.^40–42^ Further analysis of the DEGs revealed that, among the top 10 DEGs, only CFB directly participated in complement activation and served as a key molecule in this process (Figure 5(c)). This finding was further validated by qRT-PCR, which showed elevated CFB mRNA levels in M28-trained cells (Figure 5(d)). Additionally, the expression of Complement 3 (C3) was significantly increased in M28-trained macrophages, whereas no significant changes were observed in Complement 5 (C5) expression (Figure 5(d)). Treatment with CFB inhibitors (Iptacopan) decreased the release of NO and TNF-α, as well as reduced phagocytosis and killing of *S. aureus* (Figure 5(e-h)). These findings indicated that CFB played a critical role in M28-induced trained immunity.

Macrophages were pretreated with PBS or M28 (5 µg/mL) for 24 hours. After 24 hours, the cells were washed twice with PBS to remove M28 and continued incubation was performed. After 3 days, macrophages were harvested for RNA extraction and subjected to RNA sequencing (RNA-seq). (a) Volcano plot of RNA-Seq analysis (M28 group vs PBS group). (b) The top significantly up-regulated KEGG pathways in M28-trained macrophages. (c) Top 10 significantly regulated genes in M28-trained macrophages. (d) The macrophages were trained with M28 (5 µg/mL) for 24 hours and rested for 3 days. qRT-PCR quantification of complement gene expression (*n* = 3). After pretreatment with Iptacopan (CFB inhibitor) and SB290157 (C3aR inhibitor) for 24 hours, cells were treated with M28 (5 µg/mL) for an additional 24 hours. The cells were then washed twice with PBS to remove M28, and incubation was continued. 5 days later, macrophages were stimulated with LPS (100 ng/mL) for 24 hours. Supernatants were collected to measure NO (e, j) and TNF-α (f, k) levels. Alternatively, macrophages were infected with *S. aureus* (MOI = 5). Phagocytosis (g, i) and intracellular killing (h, m) of *S. aureus* were assessed at 1 and 3, 6 hours post-reinfection (*n* = 3). (i) qRT-PCR quantification of complement receptor gene expression (*n* = 3). (n) Western blot analysis of HIF-1α protein expression in M28-trained macrophages 3 days after inhibition of CFB (Iptacopan, 10 µM) or C3aR (SB290157, 10 µM). Data were presented as means ± SEM. One-way ANOVA and two-way ANOVA with Tukey’s multiple comparisons’ tests, and unpaired Student’s t tests were performed. *ns*, not significant, *P < 0.05, **P < 0.01, ***P < 0.001.

C3 is cleaved by CFB into C3a and C3b, where C3a interacts with the C3aR receptor, and C3b binds to CD46.^40^ qRT-PCR analysis revealed that M28 treatment significantly increased C3aR1 expression in macrophages compared to the PBS group, while no significant differences were observed in CD46 and C5aR1 expression levels (Figure 5(i)). Treatment with the C3aR antagonist diminished M28-induced trained immunity, as evidenced by reduced production of NO and TNF-α, along with decreased phagocytic and bactericidal capacities against *S. aureus* (Figure 5(j-m)). Additionally, HIF-1α levels decreased following the inhibition of both CFB and C3aR (Figure 5(n)). However, inhibiting HIF-1α did not affect CFB expression (Figure S6). These findings suggested that the CFB-C3a-C3aR-HIF-1α axis is the critical determinant of M28-induced trained immunity.

### M28-induced trained immunity promotes phosphatidylcholine accumulation

**Figure 6.**
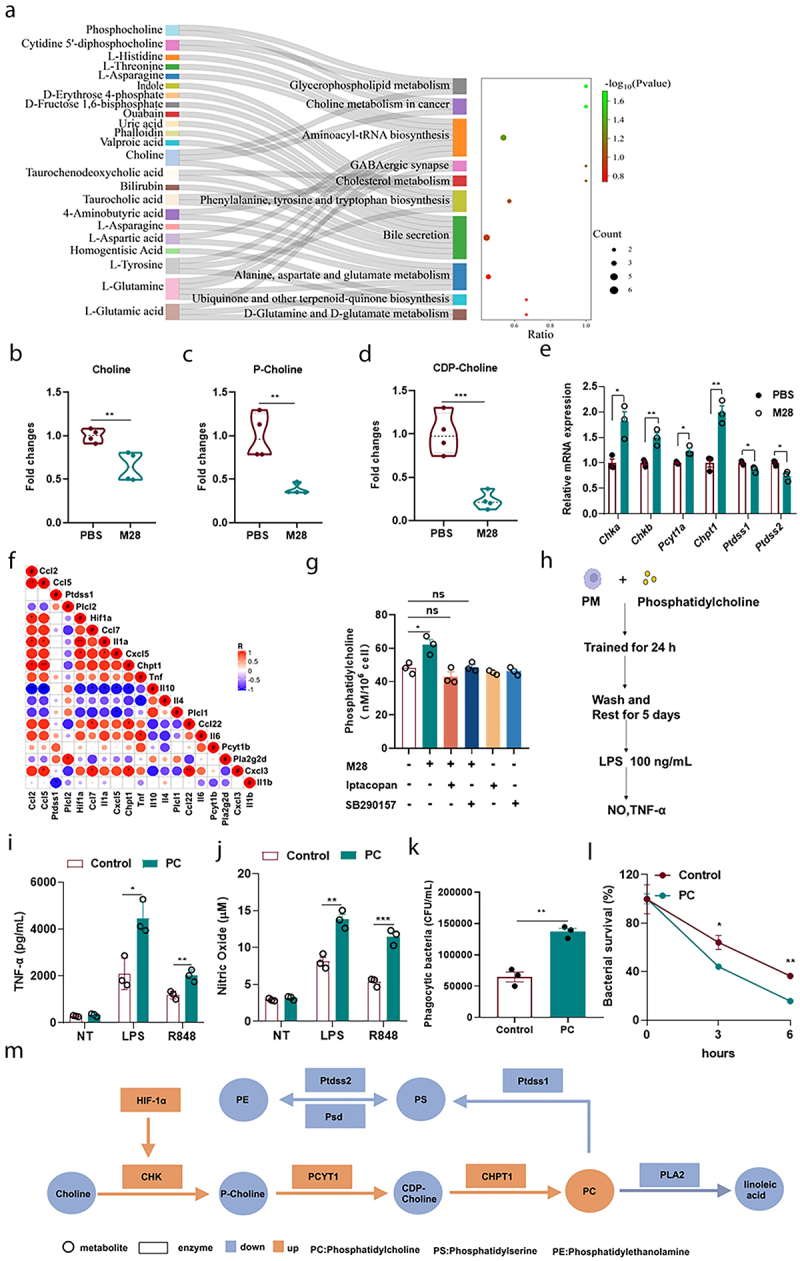
M28-induced trained immunity promotes phosphatidylcholine accumulation.

HIF-1α is a key metabolic regulator that governs various metabolic pathways, including glycolysis.^39^ The above results demonstrated that glycolysis is not a critical factor in M28-induced trained immunity (Figure 4(h-k)). Differential metabolites (DMs), KEGG enrichment analysis [variable importance in projection (VIP) > 1, *p* < 0.05] of identified differential metabolites revealed several altered pathways, including glycerophospholipid metabolism, choline metabolism in cancer, GABAergic synapse, and cholesterol metabolism. Notably, choline metabolism in cancer and glycerophospholipid metabolism were the most significantly affected pathways (Figure 6(a)). Furthermore, GSEA clustering confirmed the phenomenon (Figure S7A). The levels of key metabolites, including choline, phosphocholine, and CDP-choline, were significantly reduced following M28 treatment compared to the PBS group (Figure 6(b-d)). Further analysis indicated that choline did not directly induce trained immunity, and the exogenous addition of choline enhances the effects of M28-induced trained immunity (Figure S7B). Expression analysis of relevant enzymes in choline and glycerophospholipid metabolic pathways revealed that the mRNA levels of Chka, Chkb, Pcyt1a, and Chpt1 were increased, while the mRNA levels of Ptdss1, Ptdss2, Pla2, and Plc showed either a decrease or no significant changes (Figure 6(e) and Figure S7C). Further metabolomic data indicated that the content of phosphatidylcholine (PC) was increased, while the levels of PC-derived phosphatidylethanolamine (PE) and phosphatidylserine (PS) were reduced (Figure S7D). Phospholipase A2 (PLA2) is known to cleave phosphatidylcholine, generating linoleic acid, which has been linked to the induction of trained immunity.^43^ In M28-trained macrophages, linoleic acid levels were decreased, and inhibition of PLA2 did not impact M28-induced trained immunity (Figure S7E-G). These findings suggested that enhanced choline depletion and increased PC accumulation were associated with M28-induced trained immunity.

(a) KEGG enrichment results of DMs (VIP > 1, *p* < 0.05, |log_2_FoldChange| ≥ 1). Metabolite changes in the metabolome (*n* = 4). (b) Choline. (c) P-Choline. (d) CDP-Choline. (e) qRT-PCR quantification of glycerophospholipid metabolism genes expression (*n* = 3). (f) Correlation between cytokines genes and glycerophospholipid metabolism genes. (g) After pretreatment with the Iptacopan (CFB inhibitor) and SB290157 (C3aR inhibitor) for 24 hours, cells were treated with M28 (5 µg/mL) for an additional 24 hours. The cells were then washed twice with PBS to remove M28, and incubation was continued. 5 days later, macrophages were lysed to assess the phosphatidylcholine content. (h) Macrophages were pretreated with Control or phosphatidylcholine (PC, 5 mM) for 24 hours. After 24 hours, the cells were washed twice with PBS to remove PC and continued incubation was performed. After 5 days, the macrophages were stimulated with LPS (100 ng/mL) and R848 (100 ng/mL) for 24 hours. Supernatants were collected to measure TNF-α (i) and NO (j) levels. Alternatively, macrophages were infected with *S. aureus* (MOI = 5). Phagocytosis of *S. aureus* (k) and intracellular killing (l) was assessed at 1 and 3 and 6 hours post-reinfection (*n* = 3). (m) Metabolism summary chart. Data were presented as means ± SEM. One-way ANOVA and two-way ANOVA with Tukey’s multiple comparison tests and unpaired Student’s t tests were performed. *ns*, not significant, *P < 0.05, **P < 0.01, ***P < 0.001.

HIF-1α directly binds to the promoter region of Chka and promotes its expression.^44^ The previous results demonstrated that the CFB-C3a-C3aR-HIF-1α axis was critical for M28-induced trained immunity. Following treatment with inhibitors, the content of phosphatidylcholine was reduced in M28-trained macrophages (Figure 6(g)). Pearson correlation analysis revealed that the HIF-1α and Chpt1 genes were positively correlated with the expression of inflammatory cytokines (e.g., Tnf, Il1b, and Ccl3) and negatively correlated with the expression of Il4 and Il10. In contrast, the Ptdss1, Plcl1, and Pla2g2d genes exhibited the opposite correlation patterns (Figure 6(f)). This finding inspired us to further study the relationship between phosphatidylcholine and trained immunity. Our results showed that phosphatidylcholine induced trained immunity and enhanced M28-induced trained immunity (Figure 6(h-l)). In summary, M28-trained macrophages exhibited higher levels of phosphatidylcholine which acted as a strong inducer of trained immunity (Figure 6(m)).

### M28 liposomes-induced trained immunity prevents against S. aureus pneumonia in mice

Phosphatidylcholine is a crucial component in the synthesis of liposomes. To enhance the application of M28, we constructed liposomes encapsulating M28 using phosphatidylcholine and cholesterol via the thin-film hydration and extrusion method.

**Figure 7.**
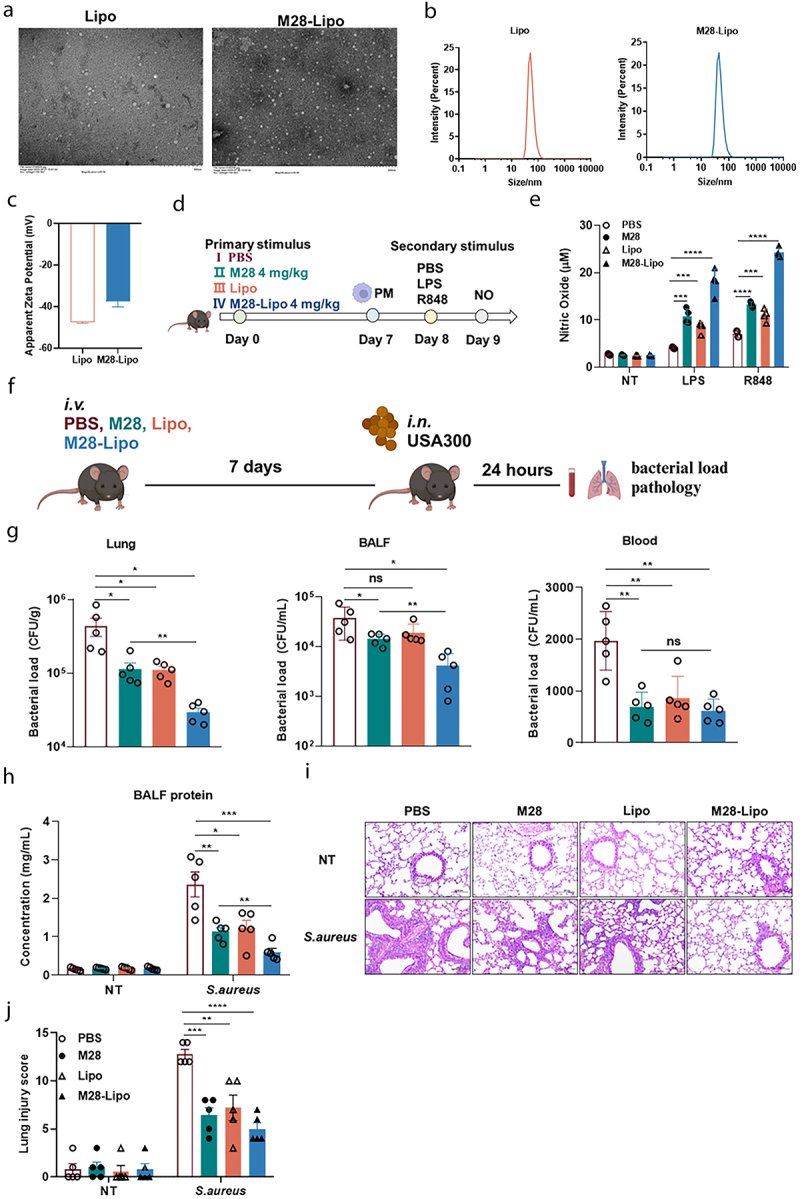
M28 liposomes demonstrate stronger resistance to *S. aureus* infection.

TEM observation revealed that both Lipo and M28-Lipo were uniformly dispersed and spherical in shape (Figure 7(a)). The particle size and zeta potential of Lipo were measured at 85.92 ± 0.35 nm and −47.5 ± 0.9 mV, respectively, while M28-Lipo exhibited a particle size of 84.41 ± 0.3 nm andzeta potential of −37.5 ± 4.7 mV with an encapsulation efficiency of 67%, and the drug loading capacity of 10.1% (Figure 7(b, c), and Figure S8A). In vitro drug release studies showed that 84% of the formulation was released within 24 hours (Figure S8B). Furthermore, utilizing the established trained immunity model of macrophages, we found that NO release in the M28-Lipo group was significantly higher than that of the PBS and M28 group restimulation with LPS. Additionally, the Lipo group also showed significantly higher NO release compared to the PBS group, which was consistent with the findings above (Figure 7(d,e)). These data suggested that M28-Lipo possessed stronger trained immune induction capabilities. Mice were treated with PBS, M28, Lipo, or M28-Lipo via tail vein injection. After 7 days, mice were nasally infected with *S. aureus* (Figure 7(f)). The results exhibited that bacterial loads in lung tissue, BALF, and blood were significantly reduced in the M28, Lipo, and M28-Lipo groups compared to the PBS group (Figure 7(g)). The PBS group exhibited more pulmonary hemorrhage, severe alveolar congestion, greater inflammatory cell infiltration, and protein leakage. Treatment with M28, Lipo, and M28-Lipo all alleviated lung tissue damage, whereas M28-Lipo treatment showed a more pronounced effect (Figure 7(h-j)). Additionally, M28-Lipo provided better protection against lethal infections caused by *S. aureus*, *Klebsiella pneumoniae*, and *Candida albicans* (Figure S8C-E). These results indicated that M28-Lipo amplified the efficacy of each component, leading to enhanced trained immunity and strengthening host defense against *S. aureus* pneumonia.

(a) Representative liposome TEM staining images (left Lipo, right M28-Lipo, scale bar: 200 nm). (b) Hydrodynamic sizes of Lipo characterized by DLS analysis. (c) Zeta potential. (d) Mice were injected via the tail vein with PBS (100 μL), M28 (4 mg/kg, 100 μL), Lipo (100 μL), or M28-Lipo (4 mg/kg, 100 μL). 7 days later, macrophages were stimulated with LPS (100 ng/mL) and R848 (100 ng/mL) for 24 hours, and supernatants were collected to measure (e) NO levels (*n* = 3). (f) Mice were injected via the tail vein with PBS (100 μL), M28 (4 mg/kg, 100 μL), Lipo (100 μL), or M28-Lipo (4 mg/kg, 100 μL). 7 days later, the mice were intranasally challenged with *S. aureus* (1 × 10^8^ CFU). 24 hours post-infection, blood, BALF, and lung tissue were collected for analysis (*n* = 5 per group). (g) Bacterial load in lung tissue, BALF, and blood (*n* = 5 per group). (h) Exudative protein concentration in BALF (*n* = 5 per group). (i) Representative H&E staining images of lung tissues from PBS and pretreated mice (scale bar: 100 μm, magnification: ×200). (J) Lung pathology scores (*n* = 5 for each group). Data were presented as means ± SEM. One-way ANOVA and two-way ANOVA with Tukey’s multiple comparisons tests and unpaired Student’s t tests were performed. ns, not significant, *P < 0.05, **P < 0.01, ***P < 0.001, **** *P* < 0.0001.

## Discussions

MRSA infections are associated with high mortality rates. It is a major cause of hospital-acquired pneumonia (HAP) and also accounts for community-acquired pneumonia (CAP) in patients.^45,46^ Vancomycin and linezolid are recommended agents in the international HAP/VAP guidelines.^47^ However, their use has been associated with adverse outcomes, including acute kidney injury and *Clostridium difficile* infection.^48^ Considering the indispensable role of innate immunity in host defense against pathogenic infections, enhancing its response may represent a potential strategy for the prevention of MRSA infection. The concept of trained immunity emerged, which was excepted to be key to solving this problem. In this study, we highlight the mechanistic role of *P. f* and its metabolite M28 in the induction of trained immunity that is causal to the protective effect of *S. aureus* infection.

A variety of microbial components have been discovered to induce the development of trained immunity or immune tolerance in innate cells. However, there are few studies exploring the ability of commensal bacteria to trigger these effects. For instance, the commensal bacterium *Lactiplantibacillus plantarum* induces innate memory-like responses in mononuclear phagocytes.^49^ The gut commensal *Limosilactobacillus reuteri*— CFS triggers an atypical memory-like phenotype in human dendritic cells in vitro.^24^ Nevertheless, no studies have yet demonstrated what types of the main effective substances mediate the above effects. The gut microbiota is involved in the host immunomodulation and microbial recognition. Bacterial metabolites or secretions constitute a vast reservoir of potential natural medicines. For a long time, natural products have played a crucial role in drug development, with soil and marine environments serving as important reservoirs of diverse compounds.^50^ Here, we found that *P. f* was a strong prior candidate for inducing trained immunity. Priming of mice or macrophages with *P. f*, especially in its live form, augmented the bacterial killing capacity and increased the release of pro-inflammatory mediators to the environment, with higher production of NO and TNF-α. Through activity tracing, we isolated the effector M28 and further demonstrated that M28 enhanced host defense against MRSA by inducing trained immunity. This represents a significant milestone for understanding the healthy symbiosis between the commensal microflora and the host.

Trained immunity, characterized by metabolic and epigenetic reprogramming of innate immune cells, has emerged as a promising area of research for adjuvant innovation. However, most studies focus on classical inducers like β-glucan or BCG, limiting the exploration of key mechanisms and novel molecular inducers of trained immunity.^51,52^ The AKT/mTOR/HIF-1α pathway plays a key role in β-glucan-induced trained immunity.^33,53^ However, the phosphorylation levels of AKT and mTOR did not change significantly, while HIF-1α expression increased in M28-induced trained immunity. Inhibition of HIF-1α significantly suppressed M28-induced training immunity. Studies have shown that C3aR deficiency can lead to reduced levels of HIF-1α and impair the C3aR-HIF-1α axis, which mediates both phagocytosis and lipid metabolism in microglia.^54^ Further analysis revealed that the CFB-C3a-C3aR axis positively regulates HIF-1α expression. The compartmentalization of complement activation and effector functions is more extensive than previously understood, occurring both extracellularly and intracellularly across various cell types and tissues.^40,55^ The intracellular complement system, known as the “complosome”, serves non-classical roles as a key regulator of essential cellular processes, such as gene transcription, mitochondrial respiration, autophagy, and glycolysis. The complosome functions in both immune and nonimmune cells, regulating normal cell turnover, responding to infectious and noninfectious stimuli, and maintaining cellular homeostatic equilibrium.^55^ In this study, we are the first to propose a link between the complement system and trained immunity. CFB cleaves C3 into C3a and C3b, with C3a binding to the C3aR receptor and activating HIF-1α, which in turn induces trained immunity. Excessive activation of the complement system is closely associated with autoimmune diseases such as systemic lupus erythematosus (SLE), a chronic, relapsing, and challenging-to-treat condition. Complement overactivation is a key contributor to tissue inflammation and damage in SLE.^56^ Similarly, the overactivation of trained immunity has been implicated in the pathogenesis of SLE.^10^ Our findings suggest that complement-mediated activation of trained immunity may underlie the recurrent flares observed in SLE. This raises the possibility that targeting complement-induced trained immunity could represent a novel therapeutic strategy. The CFB inhibitor Iptacopan, developed by Novartis, has already received approval from the U.S. Food and Drug Administration (FDA) for the treatment of paroxysmal nocturnal hemoglobinuria (PNH)^57,58^ and has been investigated in clinical trials for C3 glomerulopathy^59^ and IgA nephropathy.^60^ We hypothesize that Iptacopan could also serve as a potential therapeutic option for SLE. Further studies are warranted to explore this connection and validate the therapeutic potential of this approach.

Glycolysis, glutamine catabolism, and cholesterol synthesis pathways are known to be essential for β-glucan-induced trained immunity.^17^ However, our metabolomics data did not present significant changes in these pathways. Instead, choline metabolism in cancer and glycerophospholipid metabolism were significantly activated, leading to increased choline consumption and phosphatidylcholine accumulation, which was associated with elevated levels of HIF-1α. The glycerophospholipid-linoleic acid metabolic pathway is a crucial factor in BCG vaccine-induced trained immunity.^61^ Our results suggested that phosphatidylcholine accumulation played a central role in the induction of trained immunity. Supplementing with choline to promote phosphatidylcholine synthesis, or directly adding phosphatidylcholine, could serve as a potential strategy for enhancing immune responses through trained immunity. Excitingly, lipid nanoparticles (LNPs) encapsulating M28 with phosphatidylcholine and cholesterol significantly enhanced the capacity for trained immunity compared to M28 alone, demonstrating superior efficacy against MRSA infection. LNPs have become essential tools in drug delivery systems, particularly in the development of vaccines. The 2023 Nobel Prize in Physiology or Medicine was awarded to Katalin Karikó and Drew Weissman for their pioneering contributions to mRNA vaccine development.^62–64^ LNPs, which encapsulate modified mRNA nucleotides, have exhibited exceptional efficacy in vaccines against SARS-CoV-2, as seen in both the Pfizer (BNT162b2) and Moderna (mRNA-1273) vaccines, both of which utilize LNP carriers.^65,66^ LNP carriers are crucial in driving the adjuvanticity and immunogenicity of mRNA-LNP vaccines. This is largely attributed to their ionizable lipid components and their role in inducing the interleukin-6 (IL-6) cytokine, both of which significantly contribute to their adjuvant activity.^67,68^ Our findings further underscore the immune-enhancing properties of LNPs, specifically highlighting phosphatidylcholine as a key factor in activating trained immunity within the innate immune system. The molecule M28, or phosphatidylcholine, shows promise as a trained immunity adjuvant with the potential to extend the duration of immune memory, making it a valuable candidate for enhancing vaccine efficacy.

We identified a commensal bacteria *P. f*-derived trained immunity agonist-M28 family peptidase, which promoted choline depletion and phosphatidylcholine accumulation through the CFB-C3a-C3aR-HIF1α axis, thereby inducing trained immunity that protected against MRSA infection. These findings provide valuable strategies for the discovery of novel immunoadjuvants and vaccine adjuvants, while also offering new insights into the mechanisms underlying trained immunity. Furthermore, this research presents promising therapeutic potential for the prevention and treatment of autoimmune diseases.

## Supplementary Material

Revised Supplementary material.doc

## Data Availability

RNA-seq data has been uploaded to the NCBI SRA (PRJNA1170183). Non-targeted metabolomics data has been uploaded to MetaboLights (MTBLS11340).
