# Supplementary material for: M28 family peptidase derived from Peribacillus frigoritolerans initiates trained immunity to prevent MRSA via the complosome-phosphatidylcholine axis
Source: Gut Microbes. 2025 Mar 30;17(1):2484386. doi: 10.1080/19490976.2025.2484386 (PMC11959922; doi:10.1080/19490976.2025.2484386)
Supplement: Revised Supplementary material.doc [file KGMI_A_2484386_SM9310.doc]

**Supplementary Table and Figures**

**Supplementary Table S1 Primer sequences for qRT-PCR**

| **Gene name** | **Sequence (5’--3')** | |
| --- | --- | --- |
| Hif-1α | F | ACCTTCATCGGAAACTCCAAAG |
|  | R | CTGTTAGGCTGGGAAAAGTTAGG |
| Hk2 | F | TGATCGCCTGCTTATTCACGG |
|  | R | AACCGCCTAGAAATCTCCAGA |
| Pfkm | F | TGTGGTCCGAGTTGGTATCTT |
|  | R | GCACTTCCAATCACTGTGCC |
| Pkm2 | F | GCCGCCTGGACATTGACTC |
|  | R | CCATGAGAGAAATTCAGCCGAG |
| Cfb | F | GAAACCCTGTCACTGTCATTC |
|  | R | CCCCAAACACATACACATCC |
| C3 | F | GGCAAGACAGTCGTCATCCT |
|  | R | CCAAGACAAAGGCAAGATGC |
| C5 | F | GAACAAACCTACGTCATTTCAGC |
|  | R | GTCAACAGTGCCGCGTTTT |
| C3ar1 | F | TCGATGCTGACACCAATTCAA |
|  | R | TCCCAATAGACAAGTGAGACCAA |
| Cd46 | F | CCTTGGGCGTCTATGCTGAG |
|  | R | CCGTGGTAGTTCACAGGCATC |
| C5ar1 | F | TACCACAGAACCCAGGAGGA |
|  | R | GCCATCCGCAGGTATGTTAG |
| Chka | F | GGGTGGTCTCAGTAACATGCT |
|  | R | GAACCCTGGACTCACCATCTT |
| Chkb | F | AGGATGCTAAGTGCCCAGAG |
|  | R | TCACGGGACAAACGCTCAG |
| Pcyt1a | F | GATGCACAGAGTTCAGCTAAAGT |
|  | R | TGGCTGCCGTAAACCAACTG |
| Chapt1 | R | ACTGAGATCCAGGTAGCTTTAGT |
|  | F | GTAGACCCATTCTTGCCAACA |
| Ptdss1 | F | GCAGGACTCTGAGCAAGGATG |
|  | R | GGCGAAGTACATGAGGCTGAT |
| Ptdss2 | F | GGATTGCCTTTCAGTTCACGC |
|  | R | AGGTAGAAGGTGTTCAGCTCTG |
| β-actin | F | GGCTGTATTCCCCTCCATCG |
|  | R | CCAGTTGGTAACAATGCCATGT |

**
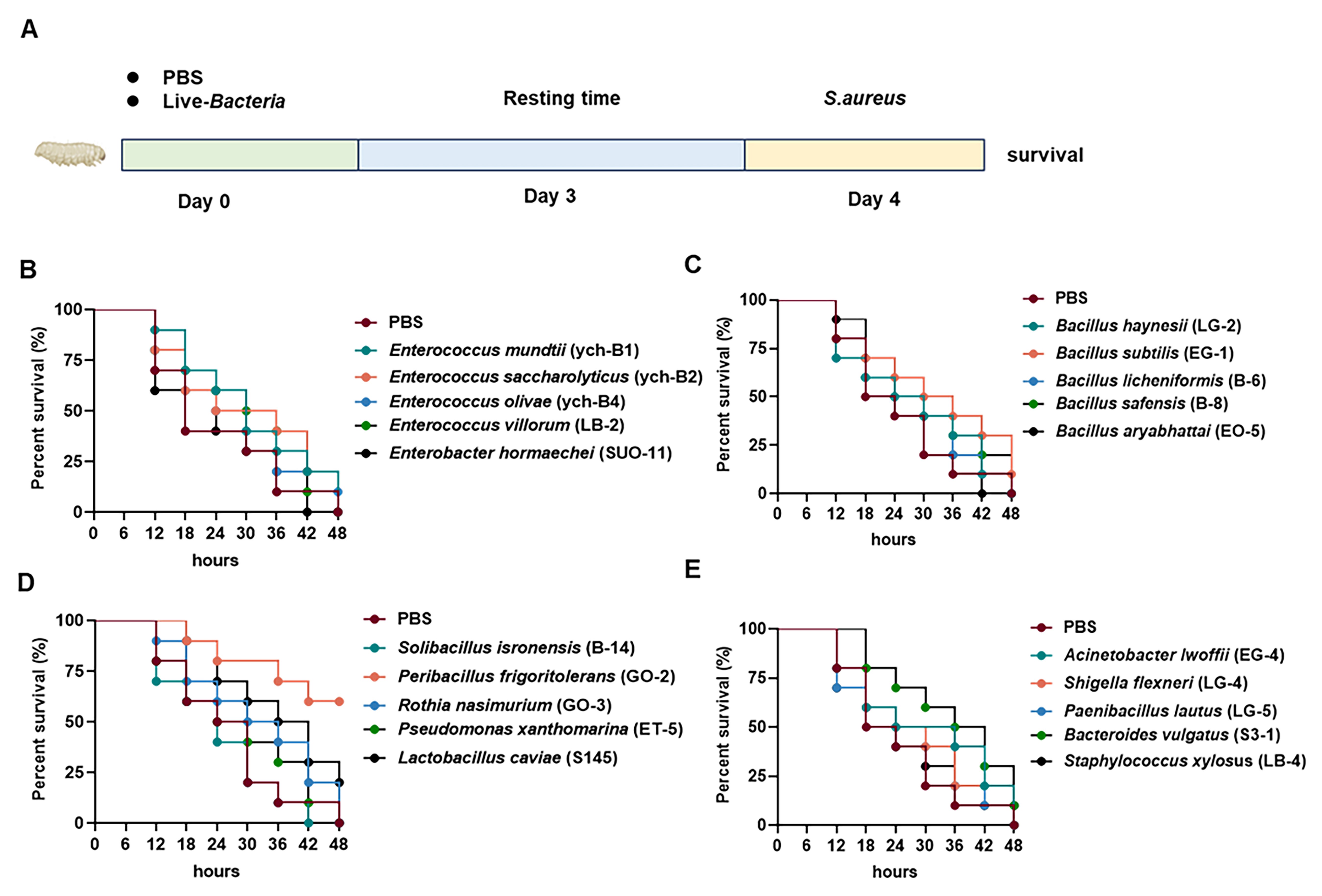
**

**Figure S1 *P. f* was identified as a potent bacteria for protection against *S. aureus* infection.**

**(A)** Schematic diagram of the *Galleria mellonella* larva experimental model. **(B-E)**. Survival rates analysis of bacteria treated larvae followed by *S. aureus* infection (n = 10 for each group). Log-rank (Mantel-Cox) tests.

**
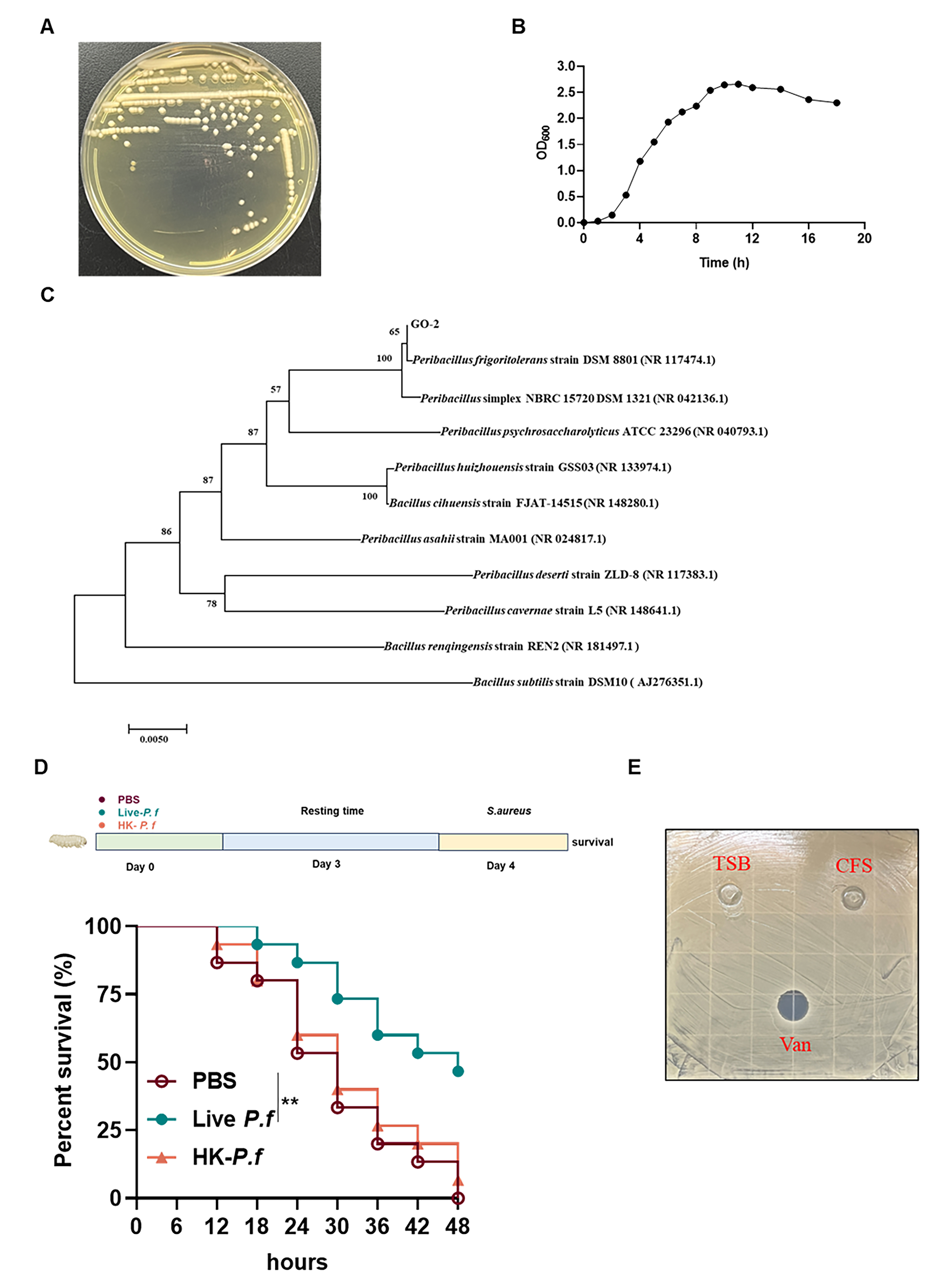
**

**Figure S2 Live *P. f* was identified as a potent strain for protection against *S. aureus* infection.**

**(A)** Colony morphology of *P. f* on TSB medium. **(B)** Growth curve of *P. f*. **(C)** Phylogenetic trees inferred from 16S rRNA gene sequences. **(D)** Schematic diagram of the *Galleria mellonella* larva experimental model (n = 10 for each group). **(E)** The inhibitory effect of *P. f* CFS (100 μL) on *S. aureus*, with vancomycin (100 μg/mL, 100 μL) as the positive control and TSB medium as the negative control. Data were presented as means ± SEM. Log-rank (Mantel-Cox) tests. ***P* < 0.01.


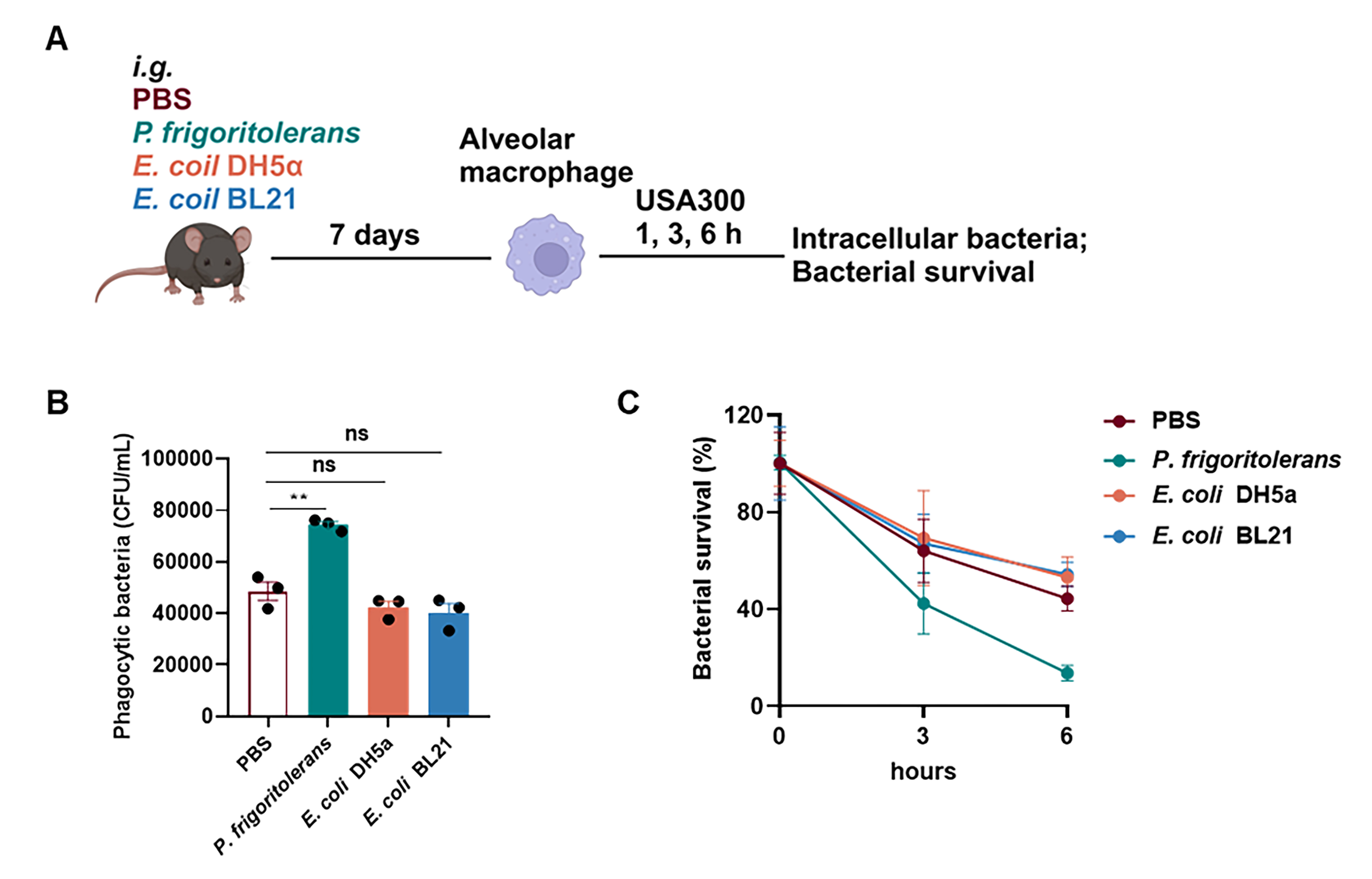


**Figure S3 Live *P. f* protects against *S. aureus* in alveolar macrophages.**

**(A)** Mice were gavaged with PBS, *P. f*, or *E. coil* DH5α, *E. coil* BL21 (Living bacteria, 1×10^8^ CFU, 200 µL) administered once daily for three consecutive days. 7 days later, macrophages were isolated from the bronchoalveolar lavage fluid and infected with *S. aureus* at a multiplicity of infection (MOI = 5), Phagocytosis **(B)** and intracellular survival **(C)** of *S. aureus* by macrophages were assessed at 1 hour or 3, 6 hours post reinfection (n = 3), Data were presented as means ± SEM. One-Way and Two-way ANOVA with Tukey's multiple comparisons tests, and unpaired Student's t tests were performed. *ns*, not significant, ***P* < 0.01.


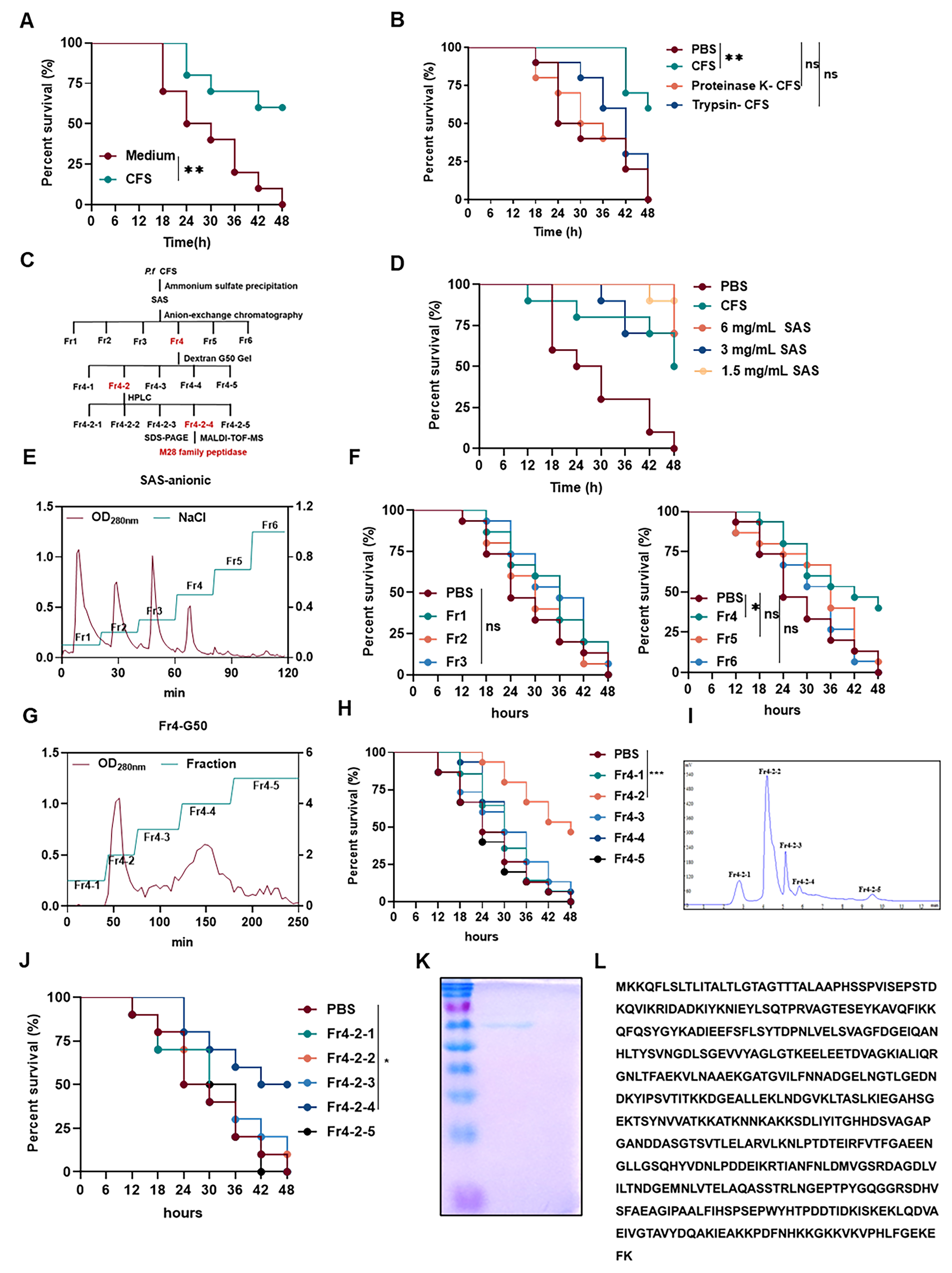


**Figure S4 The bioactive components secreted by *P. f* was isolated and identified as M28.**

**(A)** Survival rates analysis of *P. f* CFS treated larvae followed by *S. aureus* infection. **(B)** The induction activities of proteinase K and trypsin digests of *P. f* CFS. **(C)** Schematic diagram of the isolation procedure for bioactive substances produced by *P. f*. **(D)** The activities of saturated ammonium sulfate precipitate. **(E)** The ammonium sulfate precipitate was further fractionated by ion-exchange chromatography. **(F)** Survival rates analysis of components separated by ion exchange chromatography treated larvae. **(G)** The ion exchange chromatography active fraction was further separated by a G50 gel column. **(H)** Survival rates analysis of components separated by G50 treated larvae. **(I)** Further purification by preparative HPLC. **(J)** Survival rates analysis of components separated by HPLC treated larvae. **(K)** SDS-PAGE analysis of Fr4-2-4. **(L)** After SDS-PAGE analysis, the amino acid sequence of Fr4-2-4 was determined using MALDI-TOF/TOF mass spectrometry. n = 10 for each group. Log-rank (Mantel-Cox) tests. ns, not significant, **P* < 0.05, ***P* < 0.01, ****P* < 0.001.


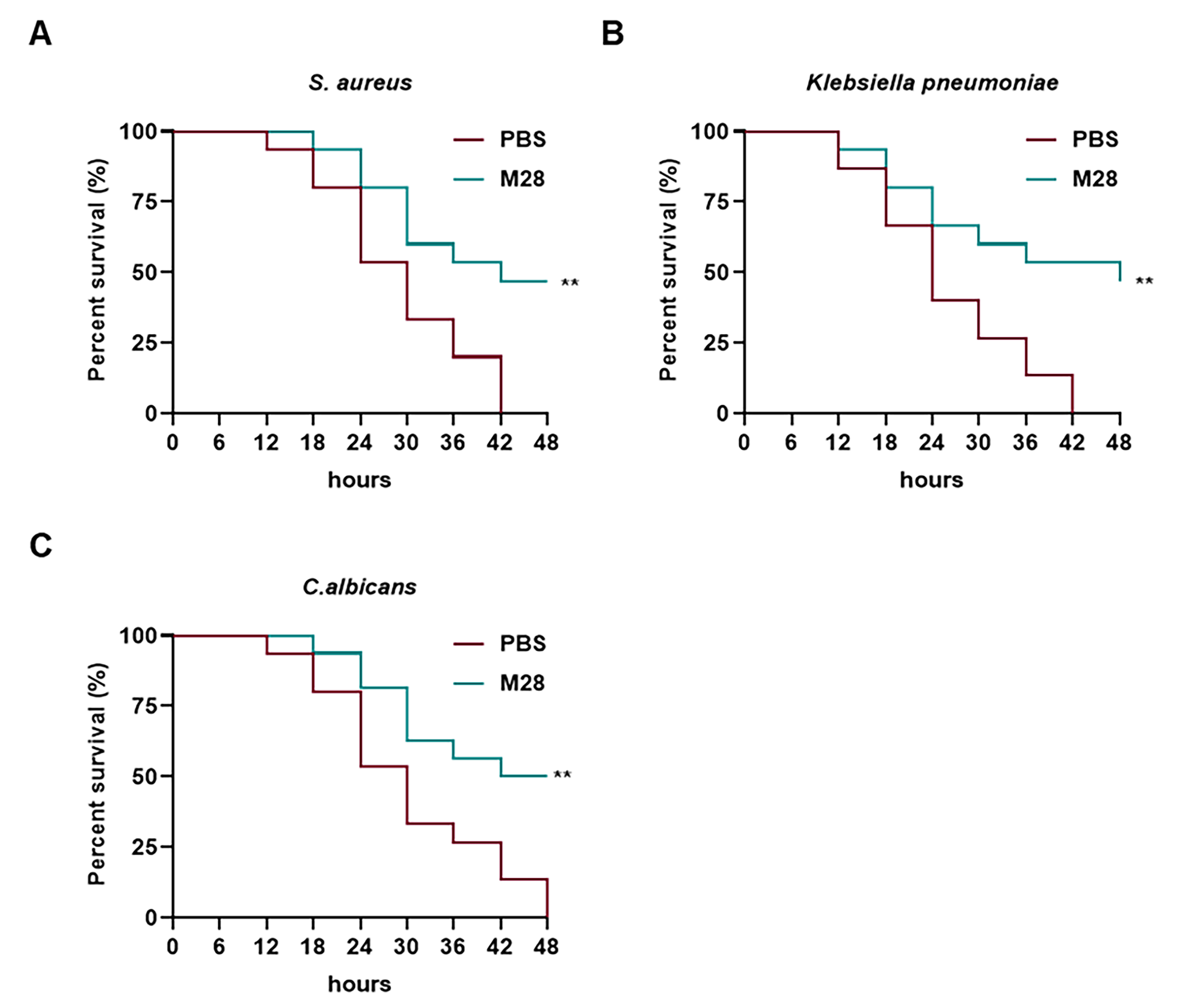


**Figure S5 M28 protects against bacteria and fungi infection.**

**(A)** Survival rates analysis of M28 (2 mg/mL, 10 μL) treated larvae followed by *S. aureus* infection. **(B)** *Klebsiella pneumoniae*. **(C)** *C. albicans*. n = 15 for each group, Log-rank (Mantel-Cox) tests. ***P* < 0.01.


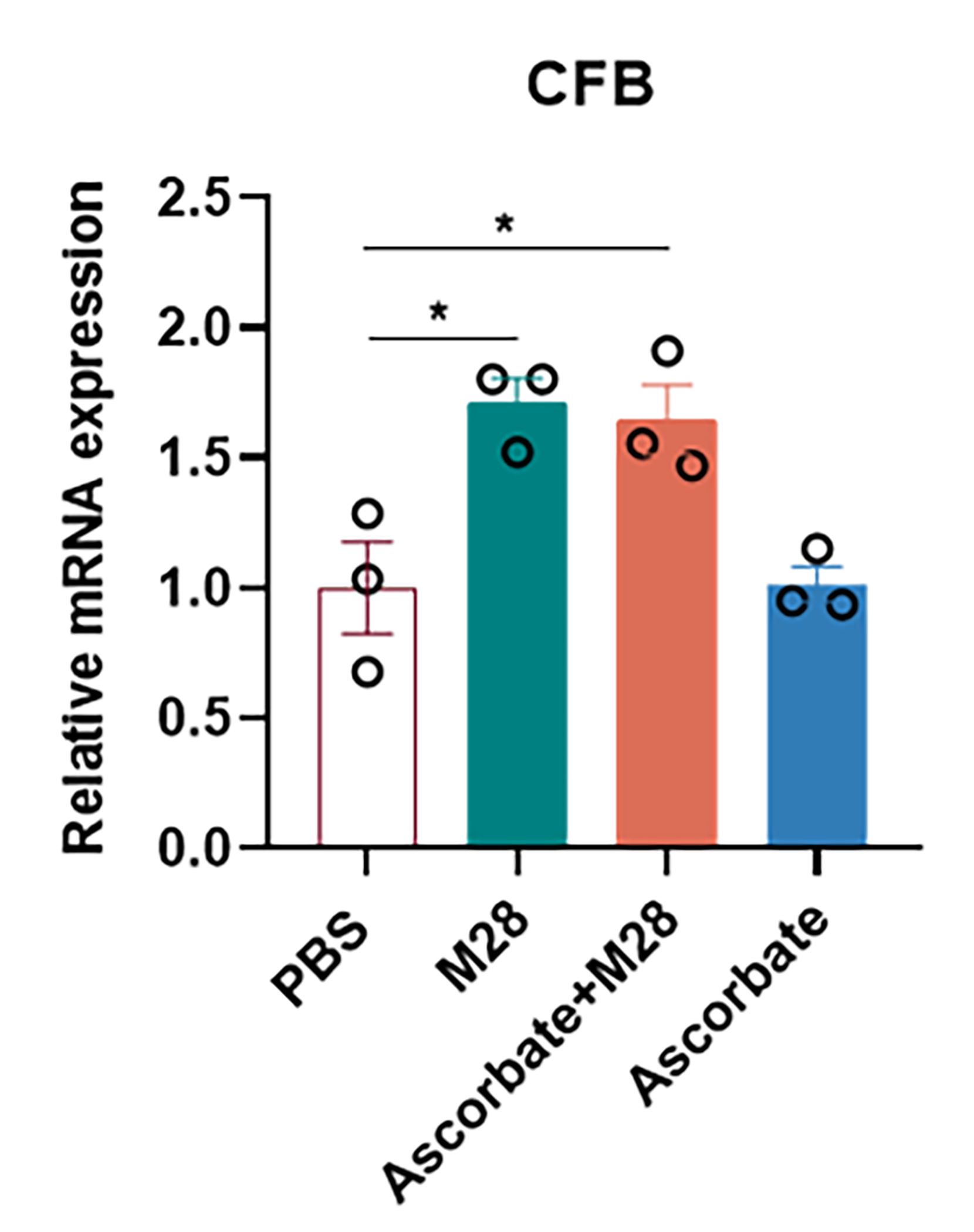


**Figure S6 Inhibition of HIF-1α does not affect CFB mRNA expression.**

For the inhibitor assay, cells were treated with ascorbate (HIF-1α inhibitor, 10 µM, pretreated for 24 h), followed by treatment with M28 (5 µg/mL) for 24 h. After this period, the cells were washed twice with PBS to remove M28, and continued incubation was performed. After 3 days, qRT-PCR quantification of CFB genes expression. Data were presented as means ± SEM (n = 3). **P* < 0.05.


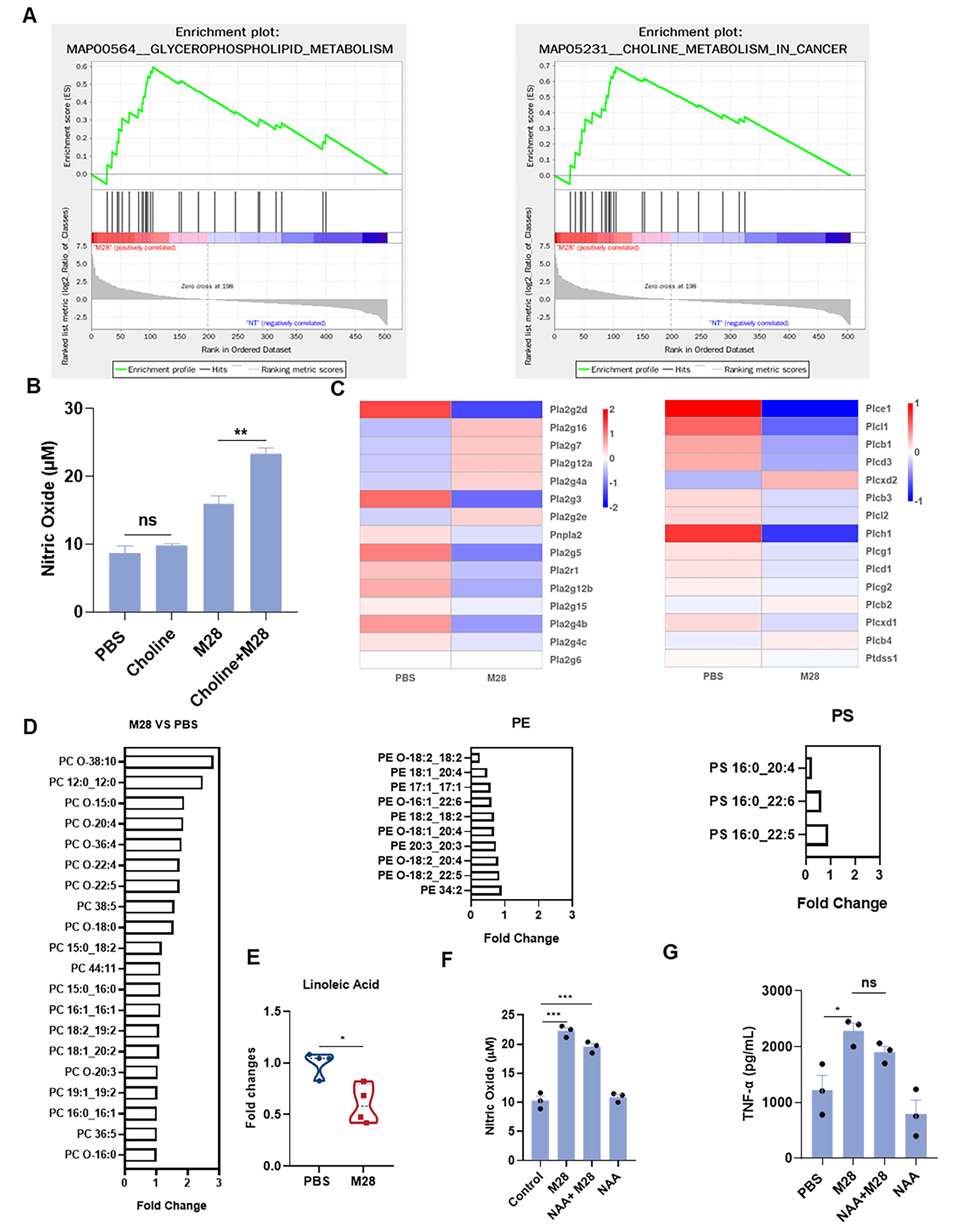


**Figure S7 M28-induced trained immunity promotes phosphatidylcholine accumulation.**

**(A)** GSEA enrichment plot of glycerophospholipid and choline in cancer metabolic pathways. **(B)** Macrophages were pretreated with PBS, choline (5 mM), or choline combined with M28 (5 µg/mL) for 24 h. After 24 h, the cells were washed twice with PBS to remove choline, and incubation was continued. 5 days later, macrophages were stimulated with LPS (100 ng/mL) for 24 h, and the supernatant was collected to measure NO levels. **(C)** Heatmap of gene expression for Pla2, Plc, and Ptdss1 in RNA-seq analysis. **(D)** Changes in phosphatidylcholine (PC), phosphatidylethanolamine (PE), and phosphatidylserine (PS) in the metabolome. **(E)** Changes in linoleic acid. After pretreatment with NAA (PLA2 inhibitor, 50 µM) for 24 h, cells were treated with M28 (5 µg/mL) for an additional 24 h. The cells were then washed twice with PBS to remove M28, and incubation was continued. 5 days later, macrophages were stimulated with LPS (100 ng/mL) for 24 h. Supernatants were collected to measure NO **(F)** and TNF-α **(G)** levels. Data were presented as means ± SEM. ns, not significant, **P* < 0.05, ***P* < 0.01, ****P* < 0.001.


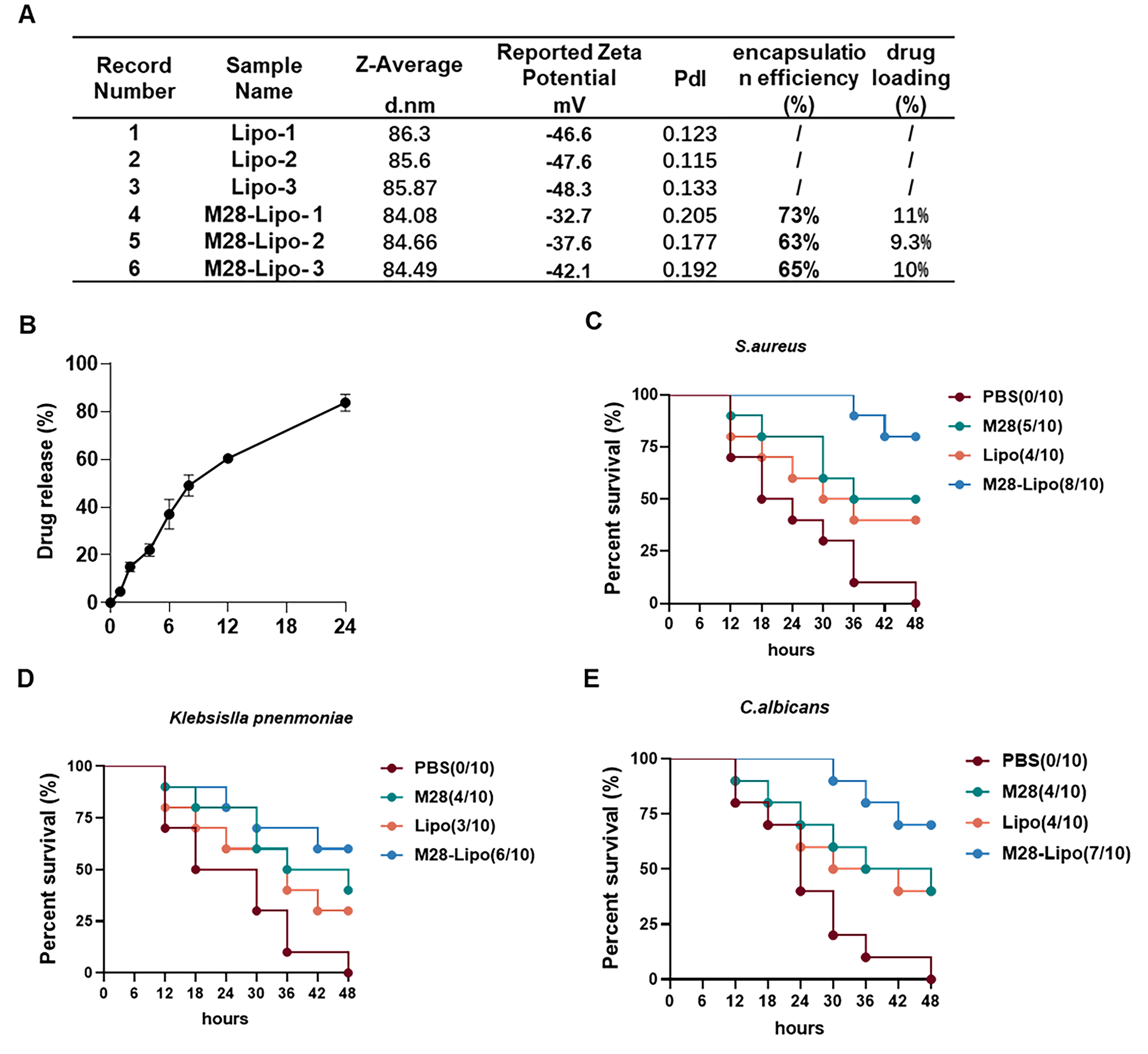


**Figure S8 M28-Lipo provides protection against bacterial and fungal infections.**

**(A)** A comprehensive analysis of M28 liposomes was performed to assess their fundamental properties. Key parameters evaluated included liposome size, polydispersity index (PDI), surface charge (zeta potential), and encapsulation efficiency. **(B)** In vitro release kinetics of M28-lipo (n = 3). **(C)** Survival rates analysis of PBS (10 μL), M28 (2 μg, 10 μL) or Lipo (10 μL), M28-Lipo (2 μg, 10 μL) treated larvae followed by *S. aureus* infection. **(D)** *Klebsiella pneumoniae*. **(E)** *C. albicans*. n = 10 for each group, Data were presented as means ± SEM. *ns*, not significant, **P* < 0.05, ***P* < 0.01, ****P* < 0.001.
